# Transport of pilgrims during Hajj: Evidence from a discrete event simulation study

**DOI:** 10.1371/journal.pone.0286460

**Published:** 2023-06-08

**Authors:** Almoaid Owaidah, Doina Olaru, Mohammed Bennamoun, Ferdous Sohel, Nazim Khan

**Affiliations:** 1 Department of Computer Science and Software Engineering, The University of Western Australia, Perth, WA, Australia; 2 Business School, The University of Western Australia, Perth, WA, Australia; 3 School of Information Technology, Murdoch University, Murdoch, WA, Australia; 4 Department of Mathematics and Statistics, The University of Western Australia, Perth, WA, Australia; Al Mansour University College-Baghdad-Iraq, IRAQ

## Abstract

Hajj, the Muslim pilgrimage, is a large mass gathering event that involves performing rituals at several sites on specific days and times in a fixed order, thereby requiring transport of pilgrims between sites. For the past two decades, Hajj transport has relied on conventional and shuttle buses, train services, and pilgrims walking along pedestrian routes that link these sites. To ensure smooth and efficient transport during Hajj, specific groups of pilgrims are allocated with the cooperation of Hajj authorities to specific time windows, modes, and routes. However, the large number of pilgrims, delays and changes in bus schedules/timetables, and occasional lack of coordination between transport modes have often caused congestion or delays in pilgrim transfer between sites, with a cascading effect on transport management. This study focuses on modelling and simulating the transport of pilgrims between the sites using a discrete event simulation tool called “ExtendSim”. Three transport modules were validated, and different scenarios were developed. These scenarios consider changes in the percentages of pilgrims allocated to each transport mode and the scheduling of various modes. The results can aid authorities to make informed decisions regarding transport strategies for managing the transport infrastructure and fleets. The proposed solutions could be implemented with judicious allocation of resources, through pre-event planning and real-time monitoring during the event.

## Introduction

Well organised transport for mass gathering (MG) events is critical for ensuring enjoyable, safe, and incident-free proceedings, regardless of the nature of the event (e.g., sporting, cultural, religious, or political) or their location. However, any MG event poses distinct challenges that are amplified by the size of the event ‎[1]. Hajj, which lasts for five days, is the largest annual MG event in the world, attracting millions of pilgrims to the sites of Makkah, Mina, Arafat, and Muzdalifah in Saudi Arabia ‎[2–5]. The pilgrims need to perform a sequence of rituals at the sites in a specific order and within a specific time window. This requires substantial crowd movements between sites ‎[6–8]. Pilgrims start their rituals at the Grand Mosque in Makkah, following which most go to their allocated camps in Mina where they spend the night (1st day of Hajj). On the 2nd day, they move (from Mina or Makkah) to Arafat to spend the whole day on Arafat Mountain, from where they must travel to reach Muzdalifah before midnight. After sunrise on the 3rd day, pilgrims return to Mina to perform the ritual of ‘Stoning the devil’ at Aljamarat Bridge; only the first of three pillars is stoned on this day. This is followed by the rituals of Tawaf and Sayee at the Grand Mosque in Makkah, and then returning to Mina. On the 4th and 5th days of Hajj pilgrims perform the ritual of stoning all three pillars at the Aljamarat Bridge. Pilgrims may now go to Makkah to perform their final Tawaf, which completes their Hajj.

The above description of Hajj provides an understanding of the complexity of issues pertaining to multiple movements of a large number of people in a short period of time, and possible overcrowding at Hajj sites. Koshak ‎[2] identified transport as the primary challenge faced by Hajj authorities which is in charge of the management and movement of pilgrims. The most frequently noted issue is the (Al-Nafrah) movement from Arafat to Muzdalifah, wherein all pilgrims need to reach Muzdalifah within a window of some six hours ‎[9–11]. Although authorities have taken measures to improve operations over the years (e.g., vehicle scheduling, monitored access to train stations, and restricting access to the sites to only to registered pilgrims and service personnel), the large number of pilgrims impacts the effectiveness and success of the transport management ‎[2, 8, 12].

Fixed and limited road networks, and the often-insufficient bus capacity relative to demand lead to congestion and traffic jams, resulting in increased travel times, bottlenecks, and affecting air quality in the entire area. Consequently, novel solutions to improve pilgrim transport between the Hajj sites have been proposed [3, 4, 13].

Currently, transport is managed and organised by the Tawafa Establishments (TEs) with the cooperation of Hajj Transport Department (Ministry of Hajj) and the Hajj Traffic Department. These establishments transport the pilgrims between the sites using designated transport modes, strictly following the transport schedules developed prior to the event [5, 8, 14].

Hajj authorities require careful prior planning to control pedestrian and vehicle movements and avoid traffic congestion and other incidents [2]. The Hajj Traffic Plan includes provisions to facilitate the movement of buses and other collective transport vehicles in Makkah [5, 8, 14]. Pilgrim scheduling is one of the measures adopted to ease congestion and avoid overcrowding [7, 8, 10, 15, 16].

Simulation studies are an important resource to inform and facilitate planning and scheduling. Despite a substantial number of studies dedicated to Hajj transport, their primarily focus is a single movement, or a single mode, or for a limited attendance, without an integration over the whole duration of the event. This paper applies Discrete Event Simulation (DES) using the “ExtendSim” software to model multiple transport movements and test scenarios. The findings are expected to provide insights to improve current transport management at Hajj, and to prepare for disruptive situations that may arise during transport of pilgrims between sites.

This paper is structured as follows. In the next section (Hajj Transport Management) we describe current Hajj transport systems and compare them with transport for other MGs events. The Review of the Simulation Literature is presented next and highlights relevant case studies and the most frequently applied transport modelling techniques. Following that, we describe our case (Data Sources) and research methodology (Modelling and Simulation Implementation), its rationale, and the description of the DES module design and validation. This is followed by a discussion of the simulation results (Model Results) and transport scenarios (Results of Scenarios), including our key findings. Finally, the paper concludes with general insights from the modelling exercise, acknowledges limitations and provides directions for future work.

## Hajj transport management

Most Hajj related studies focus on rituals as they are considered the most challenging aspects of the Hajj [7, 9, 17]. The closest events in terms of complexity and scale are large sport events such as the Olympics or the FIFA World Cup. An analysis of these two events may offer a better understanding of the common issues faced by organisers of MG events and potential solutions that can be adapted to ensure safe and efficient transport of Hajj participants.

### Hajj vs the Olympics transport management

An examination of transport management and operation during the Olympics highlights that good transport planning, a condition set by the International Olympic Committee (IOC), requires a single agency to oversee the transport system (centralised command and control) [18, 19]. This agency collaborates with government departments or authorities and private companies, which invest in infrastructure several years prior to the events to ensure adequate capacity of both accommodation and transport [18, 20–22].

The athletes are provided accommodation within specially built villages for ease of access to the sport venues and minimal travel times. Athletes and officials usually travel to the venues in buses along dedicated lanes. Visitors travel from their accommodation areas to the competition venues by point-to-point or loop mass transit systems with high frequency services. Many road projects were funded to expand existing capacity (multiple lanes, new routes or new infrastructure such as in Sydney and Athens [20, 22]). For example, in Sydney at the 2000 Olympic Games, during peak times 400,000 people per hour were transported by train. Since the year 2000, operational changes have been made in all Olympic host cities to improve and regulate traffic flow. These changes include converting streets from two-way to one-way, dedicated lanes and applying financial instruments such as tolled roads. For the Japan Olympics in 2021, technologies such as Vehicle Information and Communication System (VICS) and Advanced Mobile Information Systems (AMIS) were implemented for more effective analysis of real-time data [19, 21].

Similar substantial infrastructure changes have been implemented in Makkah, including the Aljamarat Bridge, the roads, and the introduction of the train services [23]. Yet, despite comparable numbers of participants and concentrated movements in space and time, Hajj has substantial differences in transport management compared with sport events, as highlighted in Table 1 [18].

**Table 1:** Comparison between sport events and Hajj (based on [18]).

Comparison factors	Sporting events (Olympics and World Cup)	Hajj
Frequency of the event (some location)	Every four years in different cities	Annually at Makkah city
Primary transport mode	Rail and buses	
Number of transported people	Transporting only affected participants, numbering hundreds of thousands to millions in a short time window	Transporting ALL participants, numbering in millions, in a short time window
Travel routes	Transport of affected competitors between airport, accommodation, and sports venues	Transport of ALL participants between the Hajj sites of Mina, Arafat and Muzdalifah, in a fixed order and time window, constrained by limited roads, pedestrian routes and train capacity.
Transport regulations	Dedicated bodies for transport management, including traffic monitoring, control, and central communication for efficient and real-time transport decisions.	The Ministry of Hajj, General Cars Syndicate (GCS) and the Tawaf Establishments are responsible for Hajj transport. Bus companies used for pilgrim transport must be registered to GCS systems to obtain relevant permits and comply with their rules.
Traffic operations and management	Host cities invest in Traffic Management Centres (TMC), implementing the latest Intelligent Transport Systems (ITS) to coordinate transport and especially traffic flow. Traffic restrictions may apply in certain zones. High occupancy vehicle (HOV) lanes as well as variable messaging signs are used to improve traffic flow. Multiple operational measures are applied to reduce traffic, including parking control, dedicated bus networks, promotion of public transport (PT), as well as reduced travel demand by encouraging working-from-home, extending school holidays, or supporting vacations.	Private vehicles are banned from entering the central area of Makkah and holy sites during Hajj. High occupancy vehicle lanes are not considered; instead access to sites is only by public transport (PT), walking, or park-and-ride (PnR) for locals. Several lanes are converted to one-way to enable flow of traffic. The GCS is responsible for vehicles for transport, monitoring bus movements and breakdown recovery. An electronic platform is used for traffic monitoring.
Transit operations and management	Transit operations include: • rail systems. • bus services on dedicated lanes, called "Olympic priority lanes”, to sporting venues from airports, main railway stations, accommodation, and to other city attractions. • other PT for visitors and residents to reach the sporting venues.	Transport between Hajj sites is via 2/47 buses, rail system and walking.
Public transport use	Transport is free for Olympic ticket holders, who may use any travel mode before, during and after the event.	Prior to arrival pilgrims purchase one of the PT packages offered during Hajj.

Currie and Shalaby [23] added more transport management differences between Hajj and the Olympics events to this list. While the Olympics venues change each time, Hajj venues remain fixed. This latter aspect allows planning for Hajj by accumulating knowledge, incorporating lessons from previous events in the planning of future events, incremental additions and improvements in infrastructure, and incremental improvements in procedures. Cities hosting sporting events become congested with visitors with significant crowding but not to the same extent as Hajj sites. Consequently, Hajj has often experienced dangerous conditions (such as bottlenecks, crowd turbulences and stampedes) at several holy sites, which further highlights the need for adequate planning. Currie and Shalaby [23] also pointed out the importance of education and training programs for participants and spectators for the Olympics. These are less developed for Hajj, the focus being more on group leaders who are each responsible for groups of about 250 pilgrims from the beginning to the end of Hajj.

### Management of Hajj

Hajj authorities and researchers are continually investigating innovative ways to provide better crowd and transport management strategies [11]. One of the most notable aspects of Hajj is that the Saudi government plans to ensure Hajj proceeds in a smooth manner by improving procedures and organisation in successive years. These measures are based on studies and analyses of the experience from previous years [11]. The annual process includes four phases: 1. Planning (before Hajj); 2. Operation and 3. Monitoring (during Hajj); and 4. Analysis (after Hajj). The Hajj transport management team organises and schedules the pilgrims in groups and assesses the performance of rituals, transport and accommodation using quality indicators. These indicators are adopted as the starting point, for crowd and transport modelling and simulation during this planning phase of Hajj, which feed into the Operation and Monitoring for the next Hajj.

Six Hajj authorities are involved in traffic and transport planning: the Ministry of Hajj; Traffic Police (General Security); the Ministry of Transport; GCS or Naqaba; Tawafa Establishments; and bus drivers (**Table 1**) [2]. Seven TEs are assigned to accommodate pilgrims in designated areas and organise their transport through specific modes and routes [5, 8].

The GCS organises pilgrim transport during Hajj and supervises the national transport companies. An electronic platform called “Daif” has been developed by GCS to organise Hajj transport by tracking each bus, identifying their locations and status, whether it is online, offline, moving, stopped, engine working, or suffered a breakdown [24]. This system is also used by the TEs and the transport companies to cooperate with GCS [24]. The GCS manage nearly 20,000 buses [5, 18], as described in the following section.

### Transport modes used during Hajj

**Fig 1:**
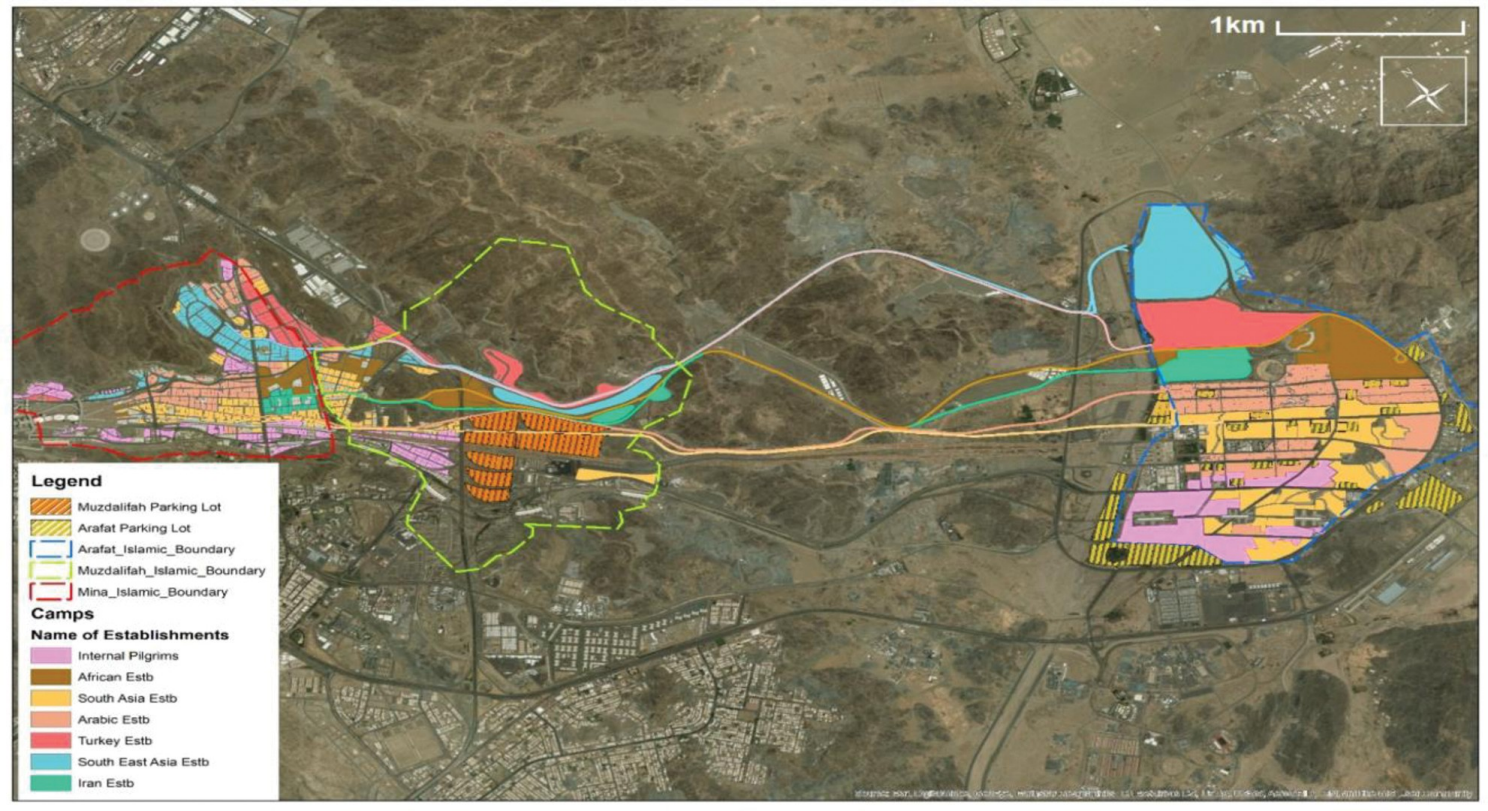
Hajj transport network (routes for buses, trains, and pedestrians) (Reprinted from [25] under a CC BY license, with permission from co-author Dr Faizan Ur Rehman, original copyright 2021). Note: The road network connecting Arafat consists of nine main roads [26, 27], of which seven, with lengths between 7 and 10 km are linked to Muzdalifah [27]. The maximum speed on these roads is 60 km/h and each has capacity 800 buses/h [26].

Pilgrim travel between the sites by buses, trains, or on foot [15]. This requires well-developed and managed transport network and services (**Fig 1**), summarised below [13].

- The three main roads in the north (light blue, lilac, and brown in (**Fig 1**) are dedicated to shuttle buses that serve four groups: Turkey, Europe, America, Australia (TEAA); Southeast Asia (SEA); Iran; and Africa.
- Trains connect the holy sites in the southern part (black lines in **Fig 1**). Where train lines intersect with roads, the railway is elevated. The trains serve four groups: the Locals; Arabic Gulf; TEAA; and South Asia.
- Pedestrian routes pass through the center of the holy sites (beige lines in **Fig 1**), connecting the Al-Namirah Mosque in Arafat and Aljamarat Bridge in Mina.
- Several roads, located north and south of pedestrian routes, are assigned to conventional buses, used to transport all pilgrim groups.

Allocation of different travel modes is based on capacity. For example, using data from the 2011 Hajj, Shihatah and Ibraheem [13] estimated that: 17% of the pilgrims (500,000) used train, a third (one million) used shuttle buses, another third (one million) used conventional buses, and the remaining 428,000 walked between the holy sites.

### Conventional and shuttle buses

Traditional (conventional) bus services make one or two trips per day to transport specific groups of pilgrims between holy sites, whereas shuttle buses make between three and nine round trips on dedicated two-way roads [28]. The primary purpose of shuttle buses is to run continuous services with fewer buses and achieve better on-time scheduling. Because the trips are much shorter, faster and not affected by congestion or interference from pedestrians, shuttle buses are also expected to reduce air pollution within and around the holy sites [29]. The trip between Arafat and Muzdalifah takes between 20 min to 195 min on a conventional bus [3], but less than 20 min on a shuttle bus. Therefore, shuttle buses ease the traffic and improve pilgrim satisfaction [6].

### Train operation

In 2009, a new railway system (Al-Mashaaer Al-Mugaddassah railway) was built to assist the transport of pilgrims between Mina, Arafat, and Muzdalifah. The railway is 20 km long and comprises nine stations (three each at Mina, Muzdalifah, and Arafat) [30]. The objective of the railway was to avoid pilgrim overcrowding, eliminate bottlenecks and heavy traffic congestion, and improve the logistics infrastructure of the holy sites [17, 27, 31]. The railway line became partially operational during the 2010 Hajj season, at below capacity [28, 30]. Currently, the railway can transport approximately 72,000 passengers/hour, equivalent to 30,000 buses [32, 33].

### Pedestrian routes between the holy sites

A substantial number of pilgrims walk between sites. **Table 2** presents details of the main pedestrian routes between the holy sites [13]. Although the distances are between 5 km and 12 km only, heavy traffic and the weather conditions (such as unbearable heat) make walking slow and pilgrims often take several hours to reach their destinations.

**Table 2:** Pedestrian routes between Hajj sites [13] (p. 10).

From	To	Direction of route	Distance (km)	Walking duration (hour)	Pilgrims per hour (p/h)
Mina	Arafat	One-way	11.62	7	69,000
Arafat	Muzdalifah		6.85	4	80,000
Muzdalifah	Aljamarat Bridge–Mina		5.38	3	40,000
Aljamarat Bridge–Mina	The Grand Mosque	Two-way	6.70	4	20,000 each direction

### Transport problems at Hajj

Owing to the large number of pilgrims, in 2019 the Hajj authorities decided to begin transport activities a day earlier than in previous year [34]. However, this proved insufficient, with statistics indicating that the scheduled transport duration was exceeded for most groups (**S1 Table**). The SEA group required 26h longer than scheduled to complete the movement from Makkah to Arafat during the first two days of Hajj. For the African group, the actual time to complete the trip was 6h more than planned.

Two exceptions are noted. The Iranian group required less time from Makkah to Mina to Arafat than planned during the first two days, but an extra hour for their trip from Makkah to Arafat on the 2nd day of Hajj. The TEAA group required 12h less on the first two days, however, this group needed an extra 7h to complete the activities on the following days and 2h more than planned to travel from Mina to Arafat.

Additional issues include breakdowns and incidents, but there were no specific data on bus breakdowns and incidents in our data for Hajj 2019 [6, 17, 35, 36]. To avoid congestion on roads caused by incidents and bus breakdowns, the “Daif” platform stores the geolocation of each bus and quickly communicates solutions to remedy incidents [24].

Given the differences between planned times and resources and those required and recorded during the event, it is both valuable and timely to develop strategies to be able to quickly alter the schedules and evaluate their impact on Hajj transport.

## Review of simulation literature

Simulation models enable us to study and understand the function or behaviour of a system over time [37]. The advantages of simulation studies include an understanding of the occurrence of a specific event, enabling the testing of hypotheses, and allowing users to experiment within complex systems. The knowledge gained could assist users in solving problems identified within systems (such as inadequate infrastructure and procedures), and most importantly, to develop scenarios for future behaviour of the system without disturbing or interfering with the real system operation or wasting resources [38]. Transport modelling can assist transport managers in planning and evaluating alternative solutions for traffic congestion by using existing networks and vehicles more effectively [3]. However, this requires time, specialised skills and appropriate software [37].

Plans for Hajj have increasingly relied on modelling and simulation of crowds and traffic [11] to understand determinants of congestion and offer suitable solutions for transport/movement between sites, thereby improving the management of operations [38]. These plans are critical because the simultaneous movement of 3–4 million pilgrims and thousands of vehicles on (spatially constrained) road networks could result in long queues and incidents [8].

Recent studies have focused on separate movements, particularly those under time constraints, such as the Al-Nafrah movement from Arafat to Muzdalifah. A 2001 study [3] highlighted that pilgrim movement between these two holy sites is a significant test for Hajj transport because it must be completed in approximately six hours. The authors developed a simulation model using the ProModel software and validated it with data from Hajj 1996. Their model suggested that 3,160 shuttle buses were required to transport pilgrims. However, the simulated number of transported pilgrims (153,000) was much smaller than that participating in Hajj. Further, the distinct composition and nationalities of the pilgrims using the buses was not included in the simulations.

**Fig 2:**
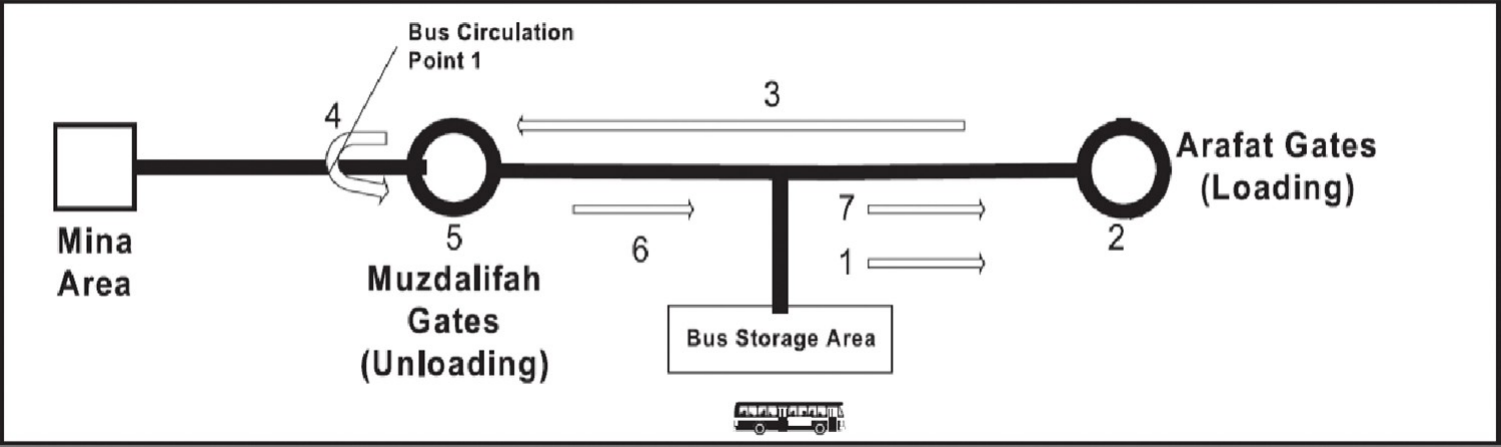
Movement 1 from Arafat to Muzdalifah (Al-Sabban and Ramadan 2005, p.76) [6].

Another study on the Al-Nafrah movement [6] also reported congestion as one of the primary traffic challenges during Hajj. The average travel time between Arafat and Muzdalifah in congestion conditions was estimated as three hours compared with only 10–15 minutes in normal traffic conditions. Therefore, the objective of [6] was to evaluate any shortcomings associated with traffic management. They built a DES model in the Arena platform using data (bus boarding, alighting and travel times and bus frequency) from the 2002 Hajj. Pilgrims from TEAA were simulated in the model (160,000 pilgrims transported by 542 shuttle buses). The authors reported that the simulation model succeeded in presenting suitable traffic management strategies for the shuttle buses as shown in **Fig 2**. Their simulation results suggested that 500+ buses were needed for the Al-Nafrah movement, which would last over 8h. However, the study simulated Hajj transport for only a single group and only two movements (Arafat to Muzdalifah and Muzdalifah to Mina) using shuttle buses and excluded conventional buses. Further, transport from Mina to Arafat was not considered.

Study [35] developed a framework consisting of intelligent agents and real-time 3D virtual environment for traffic for the Al-Nafrah movement, and modelled travel times on different roads between Arafat to Muzdalifah, offering spatial analysis of vehicle location and estimating outcomes of various traffic movement scenarios. For the validation process, the author collected data (number of buses on each road) from the Ministry of Hajj and Traffic Police, but the paper did not report the year considered for validation, the number of simulated pilgrims or their group membership. Although the paper mentioned other available transport modes (train and walking), these were not considered in their study.

A study by [36] proposed a modelling framework using Radio Frequency Identification (RFID) technology and Geographic Information Systems (GIS) to monitor and provide real-time information on 58 groups from TEAA using shuttle buses. Study [36] did not perform modelling or simulation, instead controlling, monitoring and managing Hajj transport systems using a combination of RFID and GIS relevant to the transport operation. However, the study used only one pilgrim group and did not report the year of data collection.

Study [39] highlighted the cascading effect of traffic congestion that leads to other challenges in managing Hajj, such as the late arrival of pilgrims at holy sites. The author developed scenarios of traffic movements between Arafat and Muzdalifah to optimise traffic and provide support for decision-making. The proposed Arena DES model was applied to evaluate traffic flows. The author used Google Earth images to accurately measure road distances on the network and reported movement data for the African and Iranian pilgrim groups. Though they are started at similar locations, the two groups were separated at the shuttle bus stops and travelled separately to meet again at Arafat. The in-vehicle travel time from Arafat to Muzdalifah was between 3.7 min to 5.6 min, corresponding to a speed between 40 to 60 km/h. The results of the first simulation (run time = 7.66 hours) indicated an average pilgrim waiting time of 3.42 min. As an improvement, the authors decreased the boarding and alighting times and increased the utilisation for the highways between the two holy sites. The traffic performance increased by 6%, with bus speeds reaching 65 km/h, and the average waiting time reduced to 2.9 min (model run time = 7.27 hours). However, the author did not report the source of the data used in this study, the number of transported pilgrims or the number of buses used in the simulation.

Focusing more broadly on transport management of the whole Hajj, [4] argued that many issues could affect transport between the holy sites: limited capacity compared to the demand; temporal and spatial constraints at the holy sites; and heterogeneity of the attending pilgrims and their increasing numbers every year. The most challenging task for Hajj authorities is facilitating the transport of pilgrims from Mina to Arafat and later from Arafat to Muzdalifah on the 2nd day of Hajj. Study [4] proposed an analytical and simulation-based model using the DES platform OMNeT, selected for its powerful debugging tools, detailed graphics and ability to perform sophisticated data analysis. Using this framework, the authors developed bottleneck scenarios and presented solutions for traffic management by improving bus travel times and capacity utilisation, as well as reducing congestion levels. Again, the number of pilgrims and their composition and bus types (conventional or shuttle) were not specified.

A more recent study by [40] reiterated that crowd and transport management are causes of safety concerns for the Hajj authority. This study proposed a model framework that combined GIS and agent-based modelling (ABM) to simulate movements of 66,000 pilgrims during Hajj. Two scenarios were developed: Mina to Arafat on the 2nd day; and Mina to Aljamarat Bridge on the 3rd day. Although all transport modes were included, the results were presented only for walking. Also, the number of agents simulated is small compared to the total number of pilgrims at the Hajj, and the authors argued that simulating the total number of pilgrims would require Big Data and Parallel Computing technologies.

Study [14] also highlighted that planning and predicting pilgrim movements, scheduling their movements and monitoring the ritual performances are necessary to guarantee successful Hajj management. They acknowledged the role played by the Mashaer railway project, which has substantially enhanced transport at Hajj. Trains operate at Hajj in five movements/patterns and different time windows, as follows.

- Movement A: Between Makkah and Mina during the day pre-Hajj to the early night of the 1st day, stopping at all intermediate stations.
- Movement B: Non-stop movement between Mina and Arafat from the 1st day (8 pm) to the 2nd (11 am), without stopping at any intermediate stations.
- Movement C: From Arafat to Muzdalifah from the 2nd (6:55 pm) to the 3rd (00:30 am).
- Movement D: From Muzdalifah to Mina on the 3rd from 1 am to 9 am.
- Movement E: On the last three days continuously from Mina to the Aljamarat Bridge station.

Note that there are no stations between Arafat and Muzdalifah, and between Muzdalifah and Mina. Using 2019 data [14], proposed a new schedule for the trains based on a shuttle operating pattern which included the dispatch date and time from Mina city and specified the gate at the departure station, in addition to the movement type (A, B, C, D or E), departure and arrival times, and platforms. They applied an algorithm built in MATLAB to mimic train movements in a loop. Their results showed a substantial increase in the number of pilgrims, from 304,000 to 376,000, transported between Arafat and Muzdalifah using movement C. They pointed out that following their schedules and designated paths to the station were key for this improvement. In addition, avoiding crowd build-up and preventing any incidents. However, the study did not mention details of the operation, such as travel time or number of trains used.

A related study [5] noted that crowds can cause frequent bus stoppages and road congestion during Hajj. The authors presented an interactive big data platform to visualise the movement of more than 20,000 buses during Hajj using data from the GPS trackers. This platform provides routes, travel distances, and times for each bus and each TE, and assists users to identify congestion on roads. However, no clear description was provided of how this data could be used to obtain solutions for avoiding or alleviating congestions. In addition, the solutions may differ depending on the potential users of this platform (e.g., Ministry of Hajj, GCS, or TEs), as the optimisation may reflect the different perspectives and objectives of each.

In summary, previous models and simulations for Hajj transport are insufficient and primarily address the Al-Nafrah movement. Furthermore, there are some limitations common to most models.

- Not all previous studies reported details on the year for which Hajj transport data was collected.
- With a few exceptions, previous research considered transport by bus only.
- Although the Al-Nafrah movement is critical for Hajj authorities, all pilgrim movements are important for the good organisation of Hajj and require proper planning and transport management measures in place.
- The number of pilgrims simulated and their group membership was not specified, or the numbers were too small compared to the real situation.

The present paper reports on models integrating all Hajj transport modes (buses, railways and walking), as well as all movements required during the first three days of Hajj.

We use “ExtendSim” DES as a modelling and simulation tool for Hajj transport movements. DES has been used to model a wide range of systems as connected, sequential processes [41]. Examples include arrivals for a service, resource utilisation, batching/combining resources, waiting in queues, transport and exits. We present the results of a transport model including three modules for various movements, which represents a step towards the integration of Hajj transport linking rituals at different holy sites in a single model, thus addressing a critical gap identified in the literature.

## Data sources

The lead author gathered secondary data from The Institute of Hajj and Umrah Research, and Ministry of Hajj. These included images and video material of Hajj, and tables and figures of Hajj 2019 operational planning from Hajj transport Department and The Saudi Car Syndicate Operational planning. In addition, the validation process used data from daily reports and social media coverages of Hajj 2019, corroborated with personal experiences of other co-authors from their own Hajj. These data sources were compared and cross-checked before module inputs were set, and the simulation results were compared with published statistics of Hajj and media reports.

## Modelling and simulation implementation

The model presented here is for the transport of pilgrims from The Grand Mosque to Mina (about 7 km to the East) on the 1st day of Hajj; from Mina and The Grand Mosque to Arafat on the 2nd day (20 km); and from Arafat to Muzdalifah (7 km) on the 2nd day. Mina consists of more than 100,000 tents for pilgrim accommodation and has a complex network of streets for vehicular movements [7, 42, 43]. Arafat Mountain, the most crowded site of Hajj, is an open site without transport infrastructure. Muzdalifah is a plain, level area on the route between Mina and Arafat.

**Fig 3:**
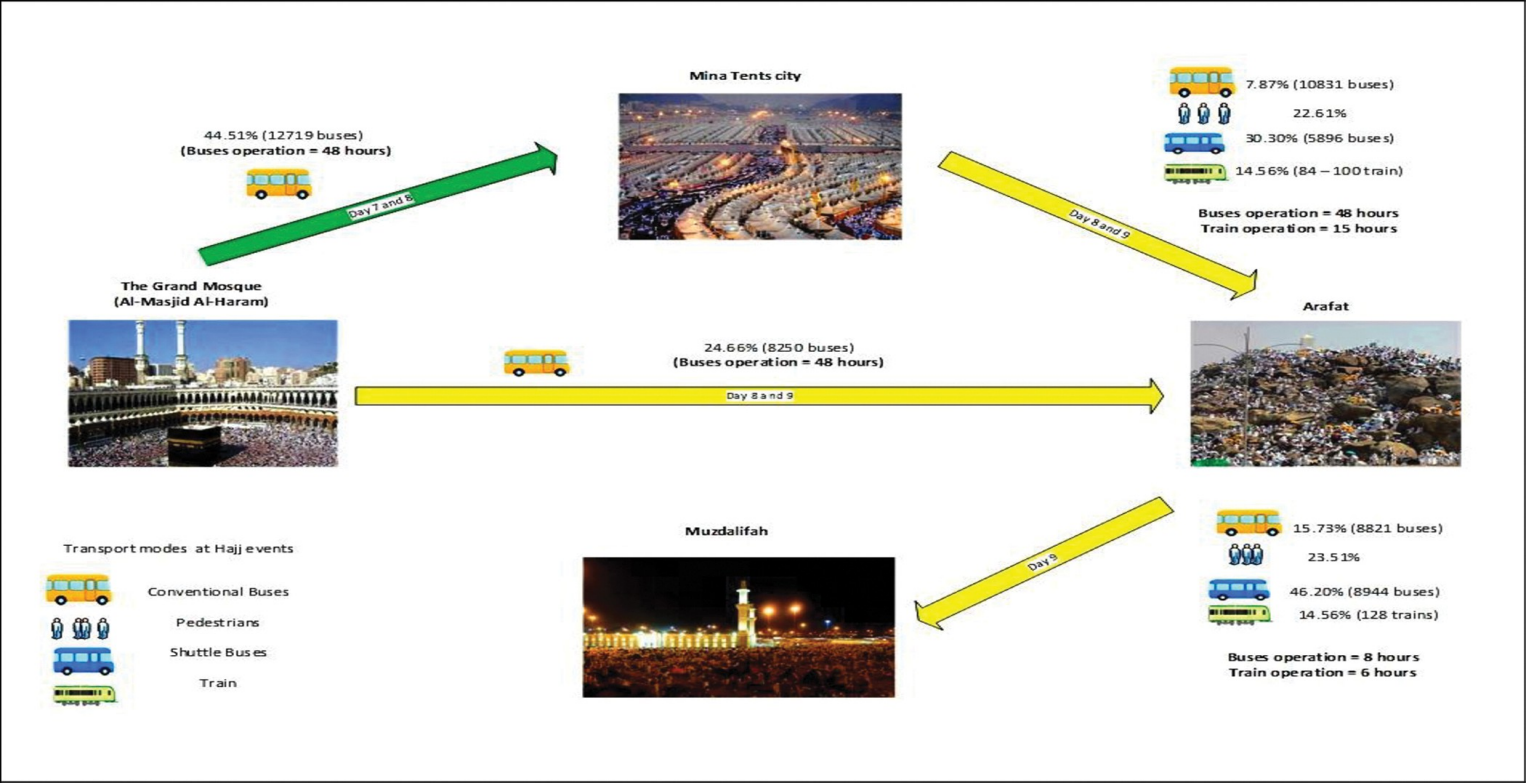
Transport operations at Hajj 2019. Note: The percentages represent the proportions of transported pilgrims by modes.

The movement schedules between these sites are as follows (**Fig 3**).

- On the 1st day of Hajj most pilgrims go to Mina and spend the night [44].
- After the night of rest, on the 2nd day of Hajj, pilgrims travel from Mina to arrive at Arafat (about 12 km) preferably before noon, but no later than sunset [44].
- Pilgrims leave Arafat Mountain for Muzdalifah as soon as possible after sunset, either walking or travelling by dedicated public transport [7].

**Fig 4:**
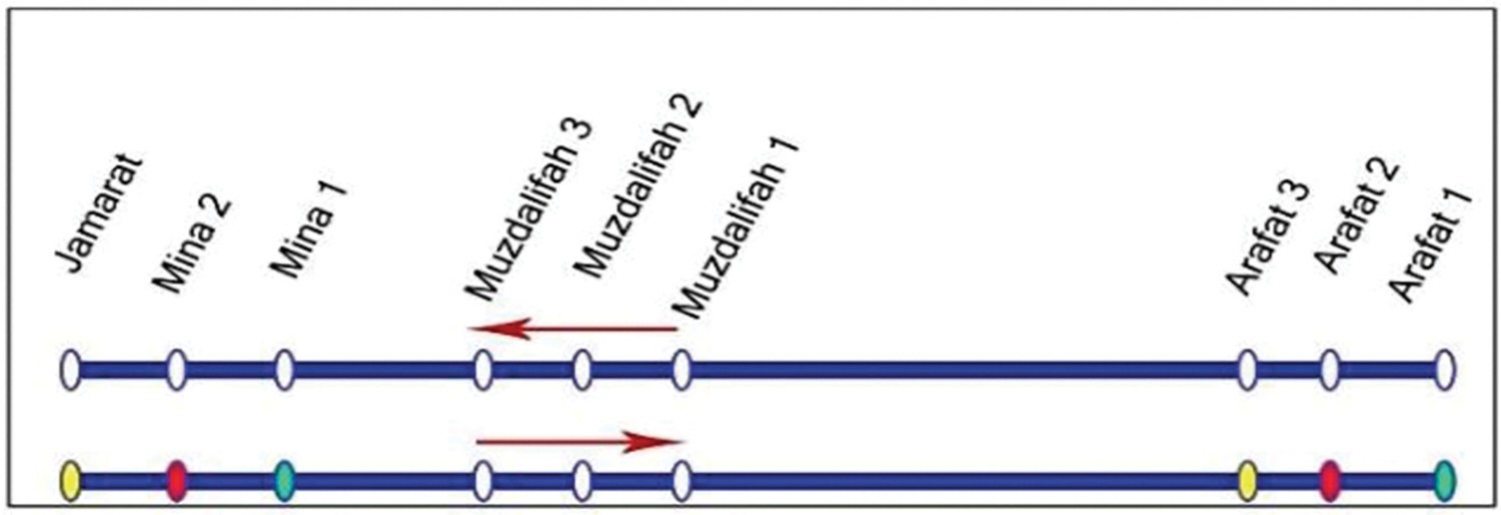
Mina-Arafat movement patterns of Al-Mashaaer Metro during Hajj (by permission from [31]). Note: Trains do not stop at Muzdalifah Stations 1, 2, and 3. The colours indicate the matching boarding-alighting stations.

The allocation of pilgrim groups to transport modes is implemented by Hajj authorities [2] to account for the fixed boundaries of the area, limited number of roads [6] and mix of traffic [3, 29], and to avoid congestion [30]. Two-thirds (more than 20,000 buses each with capacity 52 seated passengers) of the bus fleet are shuttle buses that use dedicated routes, and the rest are conventional buses. Approximately 45% of the pilgrims are transported from Makkah to Mina using conventional buses. The train operates based on specific patterns between the nine stations of the sites [30, 31] (**Fig 4**) and at any given time, there are seventeen 12-car trains running (full capacity of 20 trains), serving 3,000 pilgrims [45].

### Hajj transport model in ExtendSim

The simulation model includes sets of hierarchical blocks, each performing a specific activity. **S2 Table** presents the main blocks used in our modules and their functions. An Executive block (**S2A Fig**) located at the top left corner of each module controls the simulation timing and passage of the pilgrims through the system.

The Create block (**S2B Fig**) generates 3,000,000 pilgrims. These pilgrims have the following features incorporated in the module using Set and Get Attributes blocks (**S2C and S2D Fig**).

- **Pilgrim group and region/TE:** percentages of the total pilgrims from Hajj 2019 and their region and TE.
- **Age:** Pilgrims were categorised into two groups: 40% aged between 10 and 50 years, and 60% aged 50 years+, each with different fitness levels.
- **Speed**: Pilgrim speeds vary between 0.88 to 1.46 m/s based on their fitness and level of fatigue (three levels are assumed: rested, tired, and very tired), as mentioned in **S3 Table**. The average walking speed for the pilgrims aged 10–50 is 1.32 to 1.46 m/s, and for elderly pilgrims it is 1 to 1.2 m/s. Triangular distributions with values between 0.4 and 1.6 m/s were used to model walking speeds.
- **Fatigue**: The pilgrims’ level of energy is assumed to decrease as they progress through Hajj, and accordingly, their walking speed decreases. From "rested" to "tired," the speed drops by a factor of 1.11 and from "rested" to "very tired" it declines by a factor of 1.25. These average levels were established based on the video material and tested during the sensitivity analysis.
- **Incident rates**: Major incidents occurred at Hajj between 2002–2015 [46] involving predominantly eight countries. The incidence rates for groups more prone to accidents are found in [47]. They vary from 0.06% (Iran) to 0.53% (SA).

### Transport from Makkah to Mina (module 1)

Transport module 1 (**S2 Fig**) includes the movement of pilgrims from the Grand Mosque in Makkah to Tent City in Mina during the 1st day of Hajj. Some pilgrims stay in Makkah and travel directly to Arafat the following day (simulated in transport module 2). The numbers of pilgrims moving between the sites are listed in **Table 3** and details on the bus stops, distances from the Grand Mosque gates, and to Mina city are shown in **S4 Table** (note that pilgrim groups are mapped against bus stops accessible to them according to their location in Mina). The models assume a group of 250 pilgrims from one of the seven TEs led by a guide as the unit of analysis. of.

**Table 3:** Number of pilgrims transported by buses from site to site [34].

Pilgrims Groups	Total pilgrims’ numbers Hajj 2019	Transport Makkah to Mina (1^st^ day)		Transport Mina to Arafat (2^nd^ day)		Transport Makkah to Arafat (2^nd^ day)		Transport by train Mina to Arafat (2^nd^ day)	Transport pedestrians from Mina to Arafat (1^st^ and 2^nd^ days)	Transport Arafat to Muzdalifah (2^nd^ day)		Transport by train Arafat to Muzdalifah (2^nd^ day)	Transport pedestrians Arafat to Muzdalifah (2^nd^ day)
		# pilgrims	# buses (Conventional) [No. of trips for each bus]	# pilgrims	# buses (Shuttle and Conventional C) [No. of trips for each bus]	# pilgrims	# buses (Conventional) [No. of trips for each bus]	# pilgrims	# pilgrims	# pilgrims	# buses (Shuttle and Conventional C) [No. of trips for each bus]	# pilgrims	# pilgrims
**South East Asia (SEA)**	1,126,633	64,500	623 [2]	64,500	478 [3]	243,000	1,874 [3]	0	116,014	307,500	2,499 [3]	0	0
**South Asia (SA)**		598,158	5,773 [2]	430,615	4,680 [2]	15,283	150 [2]	167,543	0	430,615	4,680 [2]	167,543	131,297
**Iran**		11,282	68 [3]	11,282	50 [5]	78,396	477 [3]	0	0	89,678	327 [6]	0	0
**Africa**	187,814	150,394	1,003 [3]	150,394	615 [5]	0	0	0	37,420	150,394	615 [5]	0	37,420
**Arabs**	414,750	162,682	3,377 [1]	162,682	3,377 C [1]	216,953	5,085 [1]	0	35,115	370,289	7,921 C [1]	0	44,461
**TEAA**	245,002	143,263	975 [3]	143,263	731 [4]	97,537	664 [3]	0	4,202	241,906	823 [6]	0	3,096
**Arabic Gulf**	31,884	0	0	0	0	0	0	31,884	0	0	0	31,884	0
**Locals**	634,379	45,000	900 [1]	45,000	900 C [1]	0	0	185,000	403,379	45,000	900 C [1]	185,000	404,378
**Total pilgrim and buses numbers**	**2,640,462**	**1,175,279**	**12,719**	**1,007,736**	**6,554** **4277 C**	**651,169**	**8,250**	**384,427**	**597,130**	**1,635,383**	**8,944** **8,821 C**	**384,427**	**620,652**

Seven Transport blocks model the movement each of the distinct TEs, and their capacities are defined by the number of buses per hour. Based on the 2019 statistics, the module mimics the movement of approximately 1.2 million (1,175,279) pilgrims using 12,719 buses (**Table** 3) considering the number of routes available for each TE and their schedules (Shift blocks). Transport module 1 also includes seven each of Resource Pools, Batch and Unbatch blocks (**S2A–S2D Fig** respectively) used to allocate 52 passengers to a bus (Batching) and disembark them at the destination (Unbatching). The Resource Pool blocks hold a total of 14,000 conventional buses, used to complete the transport task, and split by Hajj authorities among the seven TEs. Finally, Exit blocks (**S2E Fig**) are used to pass all transported pilgrims through to the next stage of the simulation, while Plotter or Chart blocks (**S2F Fig**) are used to visualise the performance of the module.

The following simplified **Eq 1** was used in the transport block:

$$\begin{array}{l} \;(\mathrm{Traveltime}\;\mathrm{in}\;\min)\\ If(\mathrm{Incident}==0\\ \;Traveltime=\frac{1}{60}\left[\frac{\left(Access\;Distance+Egress\;Distance\right)}{\left(Speed/(ReduceSpeed\times Tired)\right)}+ttime+wait+random\right]\\ \mathrm{Else}\\ \;Traveltime=\frac{1}{60}\left[\frac{\left(Access\;Distance+Egress\;Distance\right)}{\left(Speed/(ReduceSpeed\times Tired)\right)}+ttime+wait+random\right]+10 \end{array}$$ Eq 1

where:

- ttime represents the in-vehicle travel time for the transport mode (e.g., Conventional bus travel time from Makkah to Mina is about 20 min to 40 min, depending on the route);
- Access distance: distance from the camp to the bus stop where boarding;
- Egress distance: distance from bus stop (Mina, Muzdalifah) to Al-Rahmah Mountain at Arafat area;
- Speed: pilgrims speed (**S3 Table**);
- ReduceSpeed and Tired: factors to reduce speed for elderly pilgrims and depending on the pilgrim fitness.

Table 3 also presents the numbers of pilgrims moving between the sites on the 2nd day (modules 2 and 3).

### Transport from Makkah and Mina to Arafat (module 2)

The second module includes the movement of all (3 million) pilgrims from Mina and Makkah to Arafat on the 2nd day of Hajj. Given the large number of pilgrims to be transported, all four travel modes (conventional buses, shuttle buses, trains, and walking) are used. Each motorised transport mode was modelled using Transport, Batch/Unbatch, Resource Pool, and Shift blocks using the mode distribution provided by the Hajj transport authorities (**Table 3**).

The top part of module 2 (**S4A Fig**) focuses on pilgrim movements from Mina to Arafat by shuttle buses, each of which undertakes a minimum of three return trips between these sites, and are used for only five TEs: SA, SEA, TEAA, Africa, and Iran. The Transport blocks model the movement of 30.3% of pilgrims by 5,896 shuttle buses (2019 data). The timetable for each group is set in Shift blocks. The Equation blocks feed the Transport blocks with door-to-door travel times. Except for walking, all travel times are calculated similarly to Eq 1, including access and egress to and from the station, waiting time, and in-vehicle travel time.

**S4B Fig** illustrates the transport of Arab and local (7.87%) pilgrims from Mina to Arafat using conventional buses. A total of 4,277 buses are used, each undertaking up to two round trips (2019 data). The set of blocks, **S4C Fig**, is used to model the transport of 24.66% of pilgrims directly from Makkah to Arafat without passing through Mina, using 8,100 conventional buses. This mode was applied to all pilgrim groups that stayed in Makkah. Transport movements were based on the Shift block timetables.

**S4D Fig** illustrates the transport of pilgrims from Mina to Arafat by train using the three main railway stations at each site. The Transport block for the train is set to carry 3,000 pilgrims using 128 trains per hour. In 2019, 14.56% of the pilgrims used this mode. According to the Hajj planning, only local, Arabic Gulf, TEAA, and SA pilgrims are allocated this mode of transport. More details of the train operation between Mina and Arafat and Arafat to Muzdalifah are provided in **Table 4A and 4B**.

**Table 4:** Train operations between the holy sites.

**Train operations from Mina to Arafat**					
**Access distance and time from camps to stations (walking)**	**From**	**To**	**Train distance**	**Avg. trip time**	**Egress distance and time to Al-Rahmah Mountain)**
Avg. 1.8 km (25 min)	Mina 1	Arafat 1	11.2 km	11 min	2.4 km (30 min)
Avg. 1.2 km (19 min)	Mina 2	Arafat 2	11.3 km	15 min	2.1 km (26 min)
Avg. 2.6 km (29 min)	Mina 3	Arafat 3	13 km	15 min	3.2 km (45 min)
**Train operations from Arafat to Muzdalifah (Muz)**					
**Access distance and time to Al-Rahmah Mountain) (walking)**	**From**	**To**	**Train distance**	**Avg. trip time**	**Egress distance and time to Muzdalifah area**
Avg. 2.4 km (30 min)	Arafat 1	Muz 1	7.8 km	14 min	3.1 km (39 min)
Avg. 2.1 km (26 min)	Arafat 2	Muz 2	8.3 km	9 min	1.8 km (23 min)
Avg. 3.2 km (45 min)	Arafat 3	Muz 3	8.5 km	10 min	2.5 km (31 min)

Finally, **S4E Fig** shows the 22.61% of pilgrims who walk, using the pedestrian routes between Mina and Arafat, and the Transport activity block is used to simulate these. **Eq 2** below gives an example calculation for the walking time, where ReduceSpeed and Tired are discrete factors reducing the walking speed under different conditions. It assumes that the walking time doubles in the case of an incident, wherein ReduceSpeed and Tired are discrete factors that reduce walking speed under various conditions.


$$\begin{array}{c} \left(\mathrm{Walktime}\;\mathrm{in}\;\min\right)\\ \mathrm{If}\;(\mathrm{Incident}==0)Walktime=\frac{Distance\times \frac{1}{60}}{\left(Speed/(ReduceSpeed\times Tired)\right)},\;Else\;Walktime=2\frac{Distance\times \frac{1}{60}}{\left(Speed/(ReduceSpeed\times Tired)\right)} \end{array}$$ Eq 2

### Transport from Arafat to Muzdalifah (module 3)

**Fig 5:**
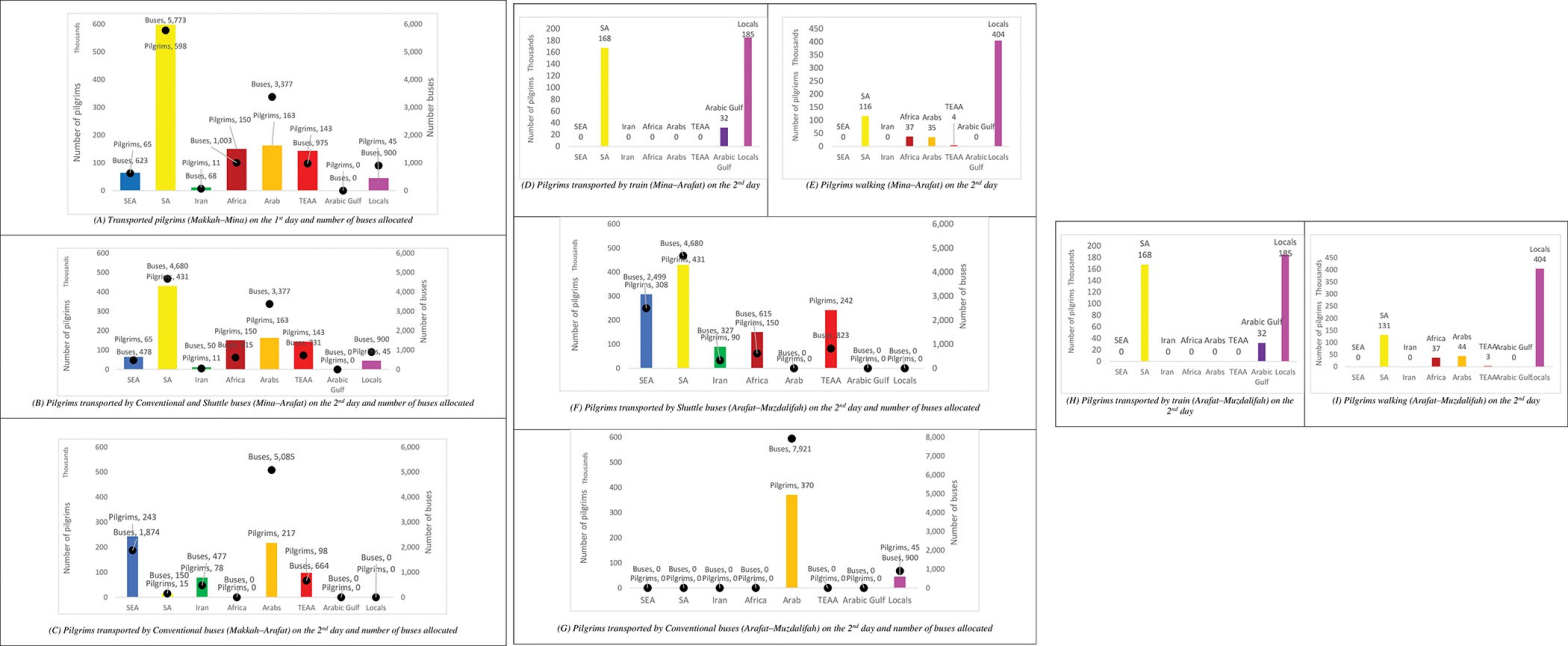
Pilgrims’ numbers and movements between the holy sites on the first two days of Hajj (Department of Transport, Ministry of Hajj, 2019). (A) Transported pilgrims (Makkah–Mina) on the 1^st^ day and number of buses allocated. (B) Pilgrims transported by Conventional and Shuttle buses (Mina–Arafat) on the 2^nd^ day and number of buses allocated. (C) Pilgrims transported by Conventional buses (Makkah–Arafat) on the 2^nd^ day and number of buses allocated. (D) Pilgrims transported by train (Mina–Arafat) on the 2^nd^ day. (E) Pilgrims walking (Mina–Arafat) on the 2^nd^ day. (F) Pilgrims transported by Shuttle buses (Arafat–Muzdalifah) on the 2^nd^ day and number of buses allocated. (G) Pilgrims transported by Conventional buses (Arafat–Muzdalifah) on the 2^nd^ day and number of buses allocated. (H) Pilgrims transported by train (Arafat–Muzdalifah) on the 2^nd^ day. (I) Pilgrims walking (Arafat–Muzdalifah) on the 2^nd^ day.

Module 3 uses the same blocks and functions of module 2, but with different Shift blocks and different train timetable. The mode share is changed, with shuttle buses increasing to 46% (8,944 buses) and conventional buses to 16% (8,821 buses). On the other hand, the railway system is increased only slightly (0.5%) and the number of pilgrims who walk is increased by 2%. All these detailed are found in **Fig 5A–5I.**

## Model results

Transport modules 1, 2, and 3 were validated with 2019 Hajj data by comparing the averages of 30 runs for each module with published statistics. **Table 5** presents the results of module 1 for travel from Makkah to Mina. For ease of presentation, group numbers were considered instead of pilgrim numbers. The simulation of the SEA and Locals groups showed the same durations as those for real data (51 min and 65 min, respectively), while the other groups recorded similar times compared to their reported aggregate data (no statistically significant differences using t-tests).

**Table 5:** Validation of module 1 (TM1)—from Makkah to Mina by conventional buses on the 1^st^ day (simulated and reported door-to-door travel times).

Mina to Arafat (Conventional and Shuttle buses)	TEAA	SEA	SA	Africa	Iran	Arabs	Locals
Number of groups*	573	260	2,430	602	45	652	180
Travel time (min) (sim results)	55	51	49	47	40	66	65
Travel time (min) (real data)	54	51	50	48	43	68	65

Note: The travel time for Locals and Arabs TE are the longest as they are allocated to the longest routes.

Results for module 2 (Mina to Arafat) are shown in **Table 6**. The simulation results compare well with the real data.

**Table 6:** Validation of module 2 (TM2)–from Makkah and Mina to Arafat by all transport modes on the 8^th^ and 9^th^ days (simulated and reported door-to-door travel times).

**Makkah to Arafat (Conventional bus)**	**TEAA**	**SEA**	**SA**	**Africa**	**Iran**	**Arabs**	**Locals**	**Arabic Gulf**
Number of groups*	390	972	61	N/A	314	868	N/A	N/A
Travel time (min) (sim results)	58	58	68		59	59		
Travel time (min) (real data)	58	56	68		59	57		
**Mina to Arafat (Conventional and Shuttle buses)**	TEAA	SEA	SA	Africa	Iran	Arabs	Locals	Arabic Gulf
Number of groups*	573	258	1,722	602	45	651	180	N/A
Travel time (min) (sim results)	54	52	61	43	51	56	64	
Travel time (min) (real data)	55	53	60	44	52	57	67	
**Mina to Arafat (Train)**	**Mina 1–Arafat 1**			**Mina 2–Arafat 2**			**Mina 3–Arafat 3**	
Number of groups*	525			496			517	
Travel time (min) (sim results)	56			56			57	
Travel time (min) (real data)	53			54			53	
**Mina to Arafat (Walking)**	**TEAA**	**SEA**	**SA**	**Africa**	**Iran**	**Arabs**	**Locals**	**Arabic Gulf**
Number of groups*	17	N/A	464	150	N/A	140	1,618	N/A
Travel time (min) (sim results)	150		145	146		145	145	
Travel time (min) (real data)	150		150	150		150	150	

Note

* Each group has 250 pilgrims.

For transport module 2, **Table 6** illustrates the results of travel from Makkah to Arafat by conventional buses, and from Mina to Arafat by shuttles and conventional buses, train, and walking. Again, the times are similar, with no statistically significant differences from the reported statistics (t-tests). One exception is the train duration from Mina stations 1, 2, and 3 to Arafat stations 1, 2, and 3, which required an extra 3–4 min compared with the reported simulation results of 53, 54, and 53 min. These minor differences are expected, as the Hajj statistics are aggregated and not reported by establishments, and details of locations in Mina city and components of door-to-door travel times were not included.

**Table 7** presents the results of the validated transport module 3 for the travel from Arafat to Muzdalifah Al-Nafrah movement) by all modes, again matching the actual times reported in 2019. The differences are minor: Iranian, African, and Arabs groups required 2 min less compared to reported statistics, whereas SA and Locals were transported in 3–4 min less than the reported statistics; transport by train 3 required 1–2 min more than the reported Hajj averages.

**Table 7:** Validation of module 3 (TM3)–from Arafat to Muzdalifah by all transport modes on the 2^nd^ day (simulated and reported door-to-door travel times).

**Arafat to Muzdalifah (Conventional and Shuttle buses)**	**TEAA**	**SEA**	**SA**	**Africa**	**Iran**	**Arabs**	**Locals**	**Arabic Gulf**
Number of groups*	968	1,230	1,722	602	359	1,481	180	N/A
Travel time (min) (sim results)	49	42	44	47	45	53	41	
Travel time (min) (real data)	49	42	47	49	47	55	45	
Arafat to Muzdalifah (Train)**	**Arafat 1–Muzdalifah 1**			**Arafat 2–Muzdalifah 2**			**Arafat 3–Muzdalifah 3**	
Number of groups*	529			467			542	
Travel time (min) (sim results)	50			51			52	
Travel time (min) (real data)	51			53			54	
**Arafat to Muzdalifah (Walking)**	**TEAA**	**SEA**	**SA**	**Africa**	**Iran**	**Arabs**	**Locals**	**Arabic Gulf**
Number of groups*	12	N/A	525	150	N/A	178	1,618	N/A
Travel time (min) (sim results)	119		115	116		115	115	
Travel time (min) (real data)	120		120	120		120	120	

Note

* Each group has 250 pilgrims.

These results provide support to the credibility of the models (as they closely reflect the real situation as reported in Hajj averages) and consequently for the results of the explored scenarios. The differences across TEs also highlight different utilisations of the fleets and reserves of capacity.

## Hajj scenarios

Transport scenarios were developed to test the system capabilities and understand how to optimally use the resources (achieve judicious allocation of buses and trains). More than 55 scenarios were tested by changing the allocated pilgrim percentages for each group and their time windows to identify the point at which the fleet was "under stress". **Table 8** lists the types and categories of transport scenarios (setups) and modules tested. The objectives were to: 1) reallocate the TEs to the four modes (change the mode share) to achieve a more consistent utilisation of the current fleets of buses and trains, while reducing the number of groups walking; and 2) to test the impact of scheduling, by re-ordering the transport of some of the TEs and compressing the timeline. Importantly, all these scenarios utilise the existing fleets, without any further investments. They also assume the same number of passengers/bus or train, thus not deteriorating the level of service by crowding the public transport.

**Table 8:** Selection of developed transport scenarios.

Module tested	Scenario type	Scenario categories	Description/Conditions
**TM1**	Scenarios related to changes in pilgrim percentages: Increasing or decreasing the pilgrim percentage allocated to different transport modes. For example, changing SEA from 20% using conventional buses and 80% using shuttles to 30% using conventional buses, 50% shuttles, and 20% walking (refer to sections Transport from Makkah and Mina to Arafat (module 2) and Transport from Arafat to Muzdalifah (module 3)).	100% Makkah to Mina	All pilgrims are travelling from Makkah to Mina on the 1^st^ day by Conventional buses (currently 44.51% of total pilgrims). No change in the bus allocation or scheduling.
**TM1**		Balanced allocation 1	Balancing the pilgrim group allocation by increasing the percentage for smaller groups and reducing it for large groups (three levels applied, 10, 20, and 30%). No change in the bus allocation.
**TM1**		Balanced allocation 2	
**TM1**		Balanced allocation 3	
**TM1**		Equal mode share 50%	Setting the same percentages of pilgrims across TEs to use a certain transport mode (50, 60, and 70%). No change in the bus allocation.
**TM1**		Equal mode share 60%	
**TM1**		Equal mode share 70%	
**TM2 and TM3**		New mode share 1	• Increasing or decreasing percentages to accommodate all transport modes. • TEAA is eligible to ride the train. • In TM3, primarily testing walking for increasing percentages of pilgrims. • No change in the bus allocation.
**TM2 and TM3**		New mode share 2	
**TM2 and TM3**		New mode share 3	
**TM1 and TM2**	Scenarios related to changes in transport timetables	Compressed time window 1	Given the relatively low utilisation of buses and trains, we tested the possibility of reducing the total time window for transport from 48 h to 36, 32 and even 24 h, to assess whether pilgrims would have more time to rest at Mina and can be transported to Arafat earlier, to reduce congestion at the site. Each timetable was organised in three separate ways and simulated within the reduced time window (original data and validated modules in 48 h): • 36 h -1: staggered, group by group. • 36 h -2: grouping large and small groups and scheduling transport in two time periods. • 36 h -3: all groups start moving at the same time (simultaneously). • No change in the bus allocation.
**TM1 and TM2**		Compressed time window 2	
**TM1 and TM2**		Compressed time window 3 –congested	
**TM1**	Rearranging bus allocation	100% Makkah to Mina -reallocation of buses	Pilgrims travelling from Makkah to Mina on the 1^st^ day by Conventional buses, while reallocating the bus numbers to each TE, but without changing the fleet size.
**TM2**		Makkah to Arafat for SA New mode share 2 and 3	Changing the bus allocations for Conventional bus mode.
**TM3**		Arafat to Muzdalifah for Locals New mode share 3	Changing the bus allocations for Conventional bus mode.

Note 1: Not all groups were eligible to use the train (SEA, Africa, Iran, and Arabs).

Note 2: This set of combinations of percentages cover the most probable alternatives that can be accepted by Hajj authorities but does not cover all possible combinations. For TMs 2 and 3, the Hajj authorities may limit access to buses.

## Results of scenarios

To avoid developing modules for unrealistic situations that are unlikely to occur during Hajj, some factors were kept unchanged. For example, we maintained the allocation of buses per TE, the routes, and the validated transport durations, focusing on the identified reserves of capacity. A similar regime was maintained for testing the 36, 32 and 24 hours (compressed time windows) scenarios developed for TM1 and TM2 (for buses), and for assessing to what extent the current 48-hour time window for pilgrims to be transported from Makkah to Mina, then from Makkah or Mina to Arafat could be compressed. However, these scenarios are not applicable for TM3, which models the Al-Nafrah movement from Arafat to Muzdalifah and has a fixed time limitation.

The first scenario of “100%” of pilgrims transported was tested only for TM1, to observe the efficiency of using the current fleet for the transport of each group without increasing the number of buses or road capacity. However, this scenario is not relevant to TM2 and TM3, because in these modules all pilgrims must use all transport modes, as planned by the Hajj authorities. The TM1 "Balanced allocation" and "Equal mode share" scenarios were conducted by changing the allocated percentage of transported pilgrims from one site to another. "New mode share" scenarios involved changing the percentage for all transport modes in TM2 and TM3.

The summary of the results is provided in **Tables 9** and **10** using the traffic-light model to indicate where problems may appear (the scenario results are available on OSF HOME website: https://osf.io/2xac8).

**Table 9:** Allocated scenarios’ results of TM1.

Scenarios TE Groups	Balanced allocation 1	Balanced allocation 2	Balanced allocation 3	Equal mode share 50%	Equal mode share 60%	Equal mode share 70%	100%
**South-East Asia (SEA)**	30%	40%	50%	50%	60%	70%	100%
**South Asia (SA)**	40%	30%	20%	50%	60%	70%	100%
**Iran**	10%	30%	50%	50%	60%	70%	100%
**Africa**	20%	30%	50%	50%	60%	70%	100%
**TEAA**	10%	20%	30%	50%	60%	70%	100%
**Arabs**	60%	50%	70%	50%	60%	70%	100%
**Locals**	10%	20%	30%	50%	60%	70%	100%

Green: Good results

Yellow: Requires more buses

Red: Requires more buses and extra time

**Table 10:** ‘New mode share’ scenario results of TMs 2 and 3 (% allocation ONLY buses)*.

TE Groups	TM2 “New mode share” scenarios	Mina–Arafat (Con and Shuttle)	Makkah to Arafat	Train	Walking	TM3 “New mode share” scenarios	Arafat–Muz (Shuttle)	Arafat–Muz (Con)	Train	Walking
**South-East Asia (SEA)**	Scenario 1	25%	50%	0%	25%	Scenario 1	60%	0%	0%	40%
	Scenario 2	35%	30%		35%	Scenario 2	40%			60%
	Scenario 3	45%	20%		35%	Scenario 3	50%			50%
**South Asia (SA)**	Scenario 1	40%	5%	10%	45%	Scenario 1	40%	0%	20%	40%
	Scenario 2	50%	10%	10%	30%	Scenario 2	50%		30%	20%
	Scenario 3	60%	15%	20%	5%	Scenario 3	60%		40%	0%
**Iran**	Scenario 1	20%	60%	0%	20%	Scenario 1	30%	0%	0%	70%
	Scenario 2	30%	70%		0%	Scenario 2	50%			50%
	Scenario 3	0%	100%		0%	Scenario 3	70%			30%
**Africa**	Scenario 1	60%	0%	0%	40%	Scenario 1	60%	0%	0%	40%
	Scenario 2	40%			60%	Scenario 2	40%			60%
	Scenario 3	50%			50%	Scenario 3	50%			50%
**Arabs**	Scenario 1	30%	40%	0%	30%	Scenario 1	0%	50%	0%	50%
	Scenario 2	50%	30%		20%	Scenario 2		40%		60%
	Scenario 3	70%	30%		0%	Scenario 3		30%		70%
**TEAA**	Scenario 1	50%	20%	10%	20%	Scenario 1	50%	0%	30%	20%
	Scenario 2	40%	50%	10%	0%	Scenario 2	40%		40%	20%
	Scenario 3	30%	30%	30%	10%	Scenario 3	30%		50%	20%
**Locals**	Scenario 1	40%	0%	30%	30%	Scenario 1	0%	5%	30%	65%
	Scenario 2	50%		20%	30%	Scenario 2		10%	40%	50%
	Scenario 3	60%		10%	30%	Scenario 3		15%	50%	35%
**Arabic Gulf**	Scenario 1	0%	0%	60%	40%	Scenario 1	0%	0%	60%	40%
	Scenario 2			40%	60%	Scenario 2			40%	60%
	Scenario 3			50%	50%	Scenario 3			50%	50%

Green: Good results

Yellow: Requires more buses/trains

Red: Requires more buses and extra scheduled time

* Note: Groups’ percentages in each scenario are complementary at all transported modes to be 100%.

### TM1—Makkah to Mina

All scenario results were compared with the validated modules. First, the "Balanced allocation" scenarios (**Table 8**) show limits of the fleet, beyond which TEs fail to satisfy the transport demand. For example, the SA transported all its groups, as they benefitted from almost 5,800 buses (see **Fig 5A**) and a substantial capacity reserve. Similarly, Africa (currently moving 80% of their pilgrims by bus) and TEAA (58% of them transported from Makkah to Mina) satisfactorily carried their groups and missed only one to two groups. However, the SEA (only 21% of them transported from Makkah to Mina in 2019, as per validated model) struggled when larger numbers were assumed to travel on the 1st day, given their allocation of 623 buses. SEA could not transport five groups for 30%, seven for 40%, and thirteen for 50% allocations. The Iranian TE effectively transported all of its groups at 10% and 30% allocations. However, when 50% of their numbers were assumed to travel from Makkah to Mina, the current small fleet of 68 buses was insufficient to carry all groups (28 groups were not transported in the time period). Arabs and Locals struggled excessively in this scenario, because of their current high utilisation of the allocated fleet (about 3,400 for Arabs and 900 for Locals, see **Fig 5A**). For the Arabs (validated for 39% of the pilgrims to travel from Makkah to Mina), when the percentage increased to above 50% and up to 70%, the allocated fleet could not complete the transport of 32 (up to 51) groups. A similar situation was encountered by the TE Locals (7% in the validated module), where the number of buses could not transport between 10 and 60 groups within the set time window when the group percentage increased from 10% to 30%.

Regarding transport durations, there were no statistically significant differences between the validated and scenario average times for the Arabs, SEA, Locals, SA, Africa, and TEAA. For the Iranian group, the first scenario resulted in a similar value to their validated duration; however, when the allocated percentage increased to 30%, the average duration increased by 16 min, and when it reached 50%, an additional 50 min was required on average to complete the transport task.

In the “Equal mode share” scenarios (50%, 60%, and 70%), a few TEs managed the tasks within the constraints with good results; for example, SA showed excellent results. However, SEA faced difficulties at these percentages and missed transporting 9–15 groups. Again, both Africa and TEAA missed transporting only two groups each. Most difficulties were encountered by the Iranian and local groups, who could not complete the transport for 99–172 and 75–111 groups, respectively. This was owing to the small number of buses that were originally allocated to these groups (see **Fig 5A**).

Yet, most groups (e.g., Arabs, SA, SEA, Africa, and TEAA) recorded average durations similar to the validated results and comparable to the 2019 Hajj, whereas Locals required an extra 2–5 min. Conversely, the Iranian group required 89 min for all scenarios, and yet it did not fully transport all of its groups in their scenario.

The worst-case scenario involved transporting “100%” of all the groups with the currently allocated fleet of buses. Unsurprisingly, SA (originally 58% transported from Makkah to Mina) was the only TE that managed carrying all of its groups, a reflection of the substantial allocation of the fleet to this group. The African (80%) and TEAA (58%) also showed good results, with only between three to six fewer groups not being transported within the time window. However, SEA and Arabs could not transport all their groups and missed 36 and 64 groups, respectively. This may be explained by the longer door-to-door travel times (15–20 min longer than most groups). Finally, as expected, the Iran and Locals TEs encountered difficulties in their transport task and could not transport 279 and 393 groups, respectively, within the time window.

For transport durations, the TEAA, SA, and Africa required an average of 2–3 min extra, whereas SEA, Iran, and Locals required 22, 90, and 75 min more, respectively.

From the above scenarios of TM1, the SA can transport up to 100%, SEA up to 40% (with extra buses), Iran up to 30%, Africa and TEAA up to 100% (with extra buses), and Arabs and Locals up to their validated percentages of 39% and 7%, respectively. With adequate bus fleet allocation, the transport task was satisfied for all groups.

The “Compressed time window” scenarios (36, 32, and 24 h) required shifting the group timetables such that they fit into the compressed timeline (all transport timetables of Conventional and Shuttle buses for Hajj 2019 and the Compressed time window scenarios of all modules are available on OSF HOME website: https://osf.io/jud8r). Compared with the allocated percentages (balanced and equal), these scenarios showed promising results for all groups except for the Locals in simulating transported groups and transport durations. However, even the group of Locals presented better results in this scenario (demand for 10–12 groups unmet, compared to 11–393 groups in the previous scenarios).

Similar results were recorded for the average travel time of all groups, apart from the Iranians and Locals (they required an average of 2–7 min and 2–4 min extra, respectively); all the other groups had similar average travel times. This suggests that distinct timetabling can enable the current fleet to satisfy the transport task by tapping into the current capacity reserves.

### TM2—Makkah and Mina to Arafat

The scenarios of TM2 were developed based on the “Compressed time window” scenarios (36, 32, and 24 h) and the “New mode share” scenarios, by distributing pilgrims between all transport modes (conventional and shuttle buses, train, and walking), as presented in **Table 10**. However, the new mode share scenarios accounted for the specific transport modes set by Hajj authorities. For example, the African group had not been transported from Makkah to Arafat, and the Arabic Gulf group was not assigned to the conventional and shuttle bus modes. Additionally, the Iranian, Africa, SEA, and Arabs were not eligible to use the train. However, the TEAA was eligible to use the train, but did not use it during the 2019 Hajj. Therefore, TEAA was added to the train scenarios.

### TM2—Makkah to Arafat by conventional buses

The “new mode share” scenarios were also applied to transport pilgrims from Makkah to Arafat (not passing through Mina) as follows: SA (5%, 10%, and 15%), SEA (20%, 30%, and 50%), Iran (60%, 70%, and 100%), TEAA (20%, 30%, and 50%), and Arabs (30%, 30%, and 40%). These scenarios showed good results by transporting most of the pilgrim groups. Iran, TEAA, Arabs, and SEA recorded similar average travel times (no significant difference from the validated module), thereby confirming that the number of vehicles allocated was sufficient, But SA required substantially more time (13 more min in the second scenario and 101 min in the third scenario). This is because this TE was allocated only 150 buses for transport (see **Fig 5C**).

The “Compressed time window” scenarios (36, 32, and 24 h) were also developed for this mode to test the ability of current transport facilities to handle each group’s transport operations. In the transport from Makkah to Arafat, only the SA completed the task within a short time interval. Additionally, the small groups of Iranians, SEA, TEAA, and Arabs could be accommodated, with only a few groups not fitting the schedule (Iran 1–5 groups, SEA 7–12 groups, TEAA 8–12 groups, and Arabs 9–13 groups). As a result, all durations were within 1–2 min of the validated average travel times.

### TM2—Mina to Arafat by conventional and shuttle buses

“New mode share” scenarios were again used in TM2, from Mina to Arafat via shuttle and conventional buses. The allocations were: SA (40%, 50%, and 60%), SEA (25%, 35%, and 45%), Iran (20%, 30%, and 40%), Africa (40%, 50%, and 60%), TEAA (30%, 40%, and 50%), Arabs (30%, 50%, and 70%), and Locals (40%, 50%, and 60%). All the groups completed the transport task. Also, these scenarios required less time than the validated duration of each TE because the tested percentages of most of these groups were lower than the validated percentages. For example, SA required 13–17 min less, while Africa, TEAA, and Arabs required 6–7 min less, and finally the Locals required 7–9 min less. However, the SEA and Iranian required an extra 1–5 min to transport their pilgrims.

For the second category of scenarios (the “compressed time window”) SA, Iran, Africa, TEAA, Arab, and Locals transported nearly all their groups, whereas the SEA transported all of them. Compared with the allocated percentages in “new mode share” scenarios, travel times for most of the groups in the reduced-time scenarios were lower by a couple of minutes, suggesting that judicious timetabling for the current module split can benefit the Hajj, by reducing the overall transport duration of the pilgrims.

### TM2—Mina to Arafat by train and walking

Three "New mode share" scenarios that were developed for the train and walking modes aimed to test the extent to which these modes can help in completing the transport of groups assigned to buses in their movements from Mina to Arafat and Makkah to Arafat. Developing scenarios that require changing transport timetables (“compressed time window”) are not applicable to train scenarios because the train is already constrained to operate within a specific time window of 15 h (which is much lower than the time windows of 36, 32, and 24 h).

In the “New mode share” scenarios, all groups were mixed and the TEAA was added to the groups of Arabic Gulf, SA, and Locals, to be transported via trains. Unlike the bus scenarios, wherein the results were reported individually per TE group, the results of the scenarios for train were calculated for the aggregated/combined TEs (see **Table 10**).

In the first “new mode share” scenario (which presented the best results), the allocated percentages were SA 10%, TEAA 10%, Locals 30%, and the Arabic Gulf 60%. Only two groups were not simulated/transported under this scenario. This means that only one extra train is needed to complete the transport of all groups (extra cars cannot be added to the trains due to platform sizes). In the second “new mode share” scenario, SA and TEAA maintained the same 10% allocation, while that of Locals was set at 20% and of Arabic Gulf at 50%. As expected, in this scenario, all groups completed their travel within the time window. However, in the third “new mode share” scenario, wherein the percentages for SA and TEAA increased to 20% and 30%, respectively, while those for Locals (10%) and Arabic Gulf (40%) diminished, 11 groups were not transported.

The duration of transport for all groups increased with the addition of TEAA. For example, Scenario 1 required an additional interval of 4–5 min, Scenario 2 required 5–6 min, and Scenario 3 required 5–8 min.

Walking scenarios also showed good results in all three allocated scenarios because all groups were able to walk from Mina to Arafat (12 km) in 150 min (2.5 h) assuming no delays. The scenarios assumed no major incidents or inclement weather conditions. Currently, there are no infrastructure limitations for the pedestrian routes.

### TM3—Arafat to Muzdalifah (Al-Nafrah movement)

The TM3 scenarios were developed based on “new mode share” scenarios, by distributing the pilgrims among all transport modes in their movement from Arafat to Muzdalifah, as presented in **Table 10**. As indicated, timetables (as per the “compressed time window” scenarios) cannot be changed owing to the time constraints imposed on the Al-Nafrah movement, which is after the sunset of the 2nd day. The results of the “new mode share” scenarios show that the transport tasks by conventional and shuttle buses were completed for all groups. In these scenarios, the percentages were as follows: SA, SEA, and Africa (40%, 50%, and 60%); Iran (30%, 50%, and 70%); and TEAA and Arabs (30%, 40%, and 50%). Moreover, except for Locals, all durations were shorter than the validated average times, explained by the larger fleets allocated to this movement (almost 18,000 buses evenly split between conventional and shuttle buses). The travel times for SA groups were shorter by 3–10 min than their validated duration, SEA by 8–10 min, Iran by 8–13 min, Africa by 3–5 min, TEAA by 7–8 min, and Arabs by 11–12 min. However, the Locals required on average an extra 2–11 min.

Similar to TM2, three “new mode share” were also developed for train, to carry the SA, TEAA, Locals, and the Arabic Gulf TEs. In the first “new mode share” scenario, the percentages were SA 20%, TEAA 30%, Locals 30%, and the Arabic Gulf 60%. Only four groups were not transported in this scenario, which required an extra train to complete the transport. The allocations in “new mode share” scenarios 2 and 3 were SA 30% and 40%, TEAA and Locals 40% and 50%, and Arabic Gulf 50% and 60%, respectively. In both scenarios, one or two extra trains were required to complete the transport. The average duration of transport increased in all train scenarios, with scenario 1 requiring an average of 3 min to complete the transport of pilgrims, whereas scenarios 2 and 3 required 5–10 min. This suggests that scenario 1 presents suitable percentages for train transport, without operational changes.

Finally, the “new mode share” scenarios for walking showed promising results for all allocated groups, as they were able to walk from Arafat to Muzdalifah (7 km) within 120 min (2 h) assuming no delays.

### Buses rearrangement for transport modules

The delays in transporting the Iranian group and the inability to complete the transport task for Arabs and Locals in TM1 suggest that fleet allocation requires adjustment. To overcome this problem, a new split of the bus fleet was proposed by rearranging the buses assigned to each group without increasing the total number of buses. The allocation was done proportional to the number of pilgrims, which means that larger TEs were allocated more buses, and the fleet allocation for small groups decreased accordingly. The final allocation was as follows: SA fleet decreased from 5,773 to 3,557, and that for Arabs decreased from 3,377 to 2,022; the fleet allocation for SEA increased from 623 to 1,499, Iran from 68 to 438, TEAA from 975 to 1,195, and Locals from 900 to 3,094; the smallest adjustment was for Africa TE, from 1,003 to 915. This new arrangement resulted in a significant improvement in bus operations from Makkah to Mina. For instance, for the Iranian group, the transport duration decreased from the validated 40 min (**Table 5**) to 35 min (10% allocation), 36 min (30%), 37 min (50%), and 38 min (60% to 70%) and only increased by 2 min for 100%. Similar results were obtained in the “100%” scenarios Makkah to Mina for almost all groups, with the transport duration of SA, Arabs and TEAA the same as their validated transport durations (49, 66 and 55 min). However, SEA and Africa required an extra one minute compared to their validated transport durations. The transport of Locals required an extra 5 min, which indicates that this group still required extra time for their scheduling to fully transport their pilgrims (**Table 5**). (The scenarios results are with the new bus numbers are available on OSF HOME website: https://osf.io/kjdf9).

Hajj 2019 bus allocation also caused difficulties in transport from Makkah to Arafat (TM2). In particular, the SA (in the “new mode share” scenarios) was unable to complete the transport of their pilgrims within the time window because of the small number of buses originally allocated (150, see **Fig 5C**). By dynamically reallocating the number of buses for each group (without changing the fleet size), the transport was improved for all TE groups. For the transport from Makkah to Arafat, the proportional allocation resulted in SA increasing their access to 1,268 buses in new share mode 2 (instead of 150, initially allocated for only 2% of the TE). In addition, in the new mode share 2, TEAA were allocated 2,128, SEA 1,602, Iran 1,090 and Arabs 2,162 (instead of 664, 1,874, 477 and 5,085 respectively–see **Fig 5C**). In new mode share 3, SA were allocated 1,969, SEA 1,106, Iran 1,614, Arabs 2,239 and TEAA 1,322 buses. Specifically for the SA group, the transport durations were reduced from 81 to 54 min and 169 to 54 min in the scenarios of “new mode share 2” and “new mode share 3” respectively, and less than their validated transport duration (68 min–see **Table 6**). This result indicates that the process of flexibly re-assigning buses based on the number of pilgrims in each group (as opposed to fixed allocation) is likely to reduce the transport duration without further increasing the fleet or altering the level of service (crowding of the buses).

The same conclusion was drawn for TM3, wherein the only group that had a mismatched allocation of bus services to the transport demand was Locals. Re-distributing the buses and allocating 1,625 buses to the Locals (the original number of buses 900 –see **Fig 5G**), reduced the transport duration by 11 min. This indicates that the transport duration and bus utilisation can be improved by allocating buses based on the number of pilgrims.

In summary, we developed a method to improve the utility of current modes of transport. Two types of scenarios were tested: 1) changing percentages of each TE group per transport mode or altering the mode in which the current capacity allocated to them is used; and 2) reducing the time window and identifying groups that can tolerate compressed timetables (36, 32, and 24 hours) for transport between the four main sites (Makkah, Mina, Muzdalifah, and Arafat).

**Tables 9** and **10** summarise all groups and their allocated percentages in each scenario, and the suitable distribution that can ensure good utilisation of transport capacity.

These scenarios considered a fixed supply of services and examined a more judicious use of existing resources; hence, the number of buses, road capacity, and train frequency were kept unchanged. Also, no changes to LoS were made, maintaining the current level of service (52 passengers/bus and 3,000 passengers/train).

From the train scenarios, the main finding is that adding a group of TEAA pilgrims would not pose any challenges to the train capacity (one or two extra trains would be required). Also, more pilgrims can be easily accommodated by approving extra time for transport by train. The results show that for TM2, the allocation for SA and TEAA can be increased by up to 10%, for Locals by 30%, and for Arabic Gulf by up to 60%. For TM3, the maximum allocation of SA without substantially changing travel times is 20%, for TEAA and Locals 30%, and 60% for the Arabic Gulf.

**Table 11** explains the current transport operations of each group and highlights situations when the capacity was inadequate. Additionally, suggested solutions are provided for each group/scenario based on the current facilities (Hajj Transport Data 2019). These solutions could be considered as alternatives for further exploration and adoption in transport operations by Hajj authorities. However, these authorities make the final decision for transport, based on their annually developed plans based on the accepted number of TE groups, fleets and routes.

**Table 11:** Suggested transport solutions for identified problems (scenarios from Tables 9 and 10).

Model	TE Groups	Transport management from Real data in the validated module			Suggested transport solutions
		Current % in the module	No. of buses	Total scheduled transport duration in the module (h)	
**TM1 Makkah to Mina**	SEA	21	623	8	• Increase number of buses and routes • Increase scheduled time. • Allocate up to 50% to be transported from Makkah to Arafat on the 2^nd^ day (Conventional mode) • Allocate up to 35% to be transported from Mina to Arafat on the 2^nd^ day (Shuttle mode)
	Iran	13	68	4	• Increase number of buses and routes • Increase scheduled time. • Allocate up to 100% to be transported from Makkah to Arafat on the 2^nd^ day (Conventional mode) • Allocate up to 30% to be transported from Mina to Arafat on the 2^nd^ day (Shuttle mode)
	Africa	80	1,003	15	• Increase number of buses and scheduled time
	TEAA	58	975	16	• Increase number of buses and scheduled time • Allocate up to 50% to be transported from Makkah to Arafat on the 2^nd^ day (Conventional mode) • Allocate up to 50% to be transported from Mina to Arafat on the 2^nd^ day (Shuttle mode)
	Arabs	39	3,377	6	• Increase number of routes• Increase scheduled time. • Allocate up to 40% to be transported from Makkah to Arafat on the 2^nd^ day (Conventional mode) • Allocate up to 70% to be transported from Mina to Arafat on the 2^nd^ day (Conventional mode)
	Locals	7	900	5	• Increase number of buses and scheduled time • Allocate up to 60% to be transported from Mina to Arafat on the 2^nd^ day (Conventional mode) • Allocate up to 30% to be transported from Mina to Arafat by Train
**TM2 Mina to Arafat**	SEA	21	478	3	• Increase number of buses and scheduled time • Allocate up to 50% to be transported from Makkah to Arafat on the 2^nd^ day (Conventional mode) • Allocate up to 35% to be transported via Pedestrian routes
**TM2 Makkah to Arafat**	SA	2	150	7	• Increase number of buses and scheduled time • Allocate up to 60% to be transported from Mina to Arafat on the 2^nd^ day (Shuttle mode) • Consider allocating additional groups to pedestrian routes (weather permitting)• Allocate up to 10% to be transported by Train
**TM3 Arafat to Muzdalifah**	Locals	7	900	7	• Increase number of buses • Consider allocating additional groups to pedestrian routes (if fitting) • Allocate up to 30% to be transported by Train

The results of this study are generally consistent with the literature; however, there are some differences in the scale of the operations described.

- Study [6] simulated 160,000 TEAA pilgrims travelling on 542 shuttle buses. In this study, all the TEAA groups (245,002 pilgrims) were simulated using various transport modes. The results of study [6] suggested using 500+ buses for the TEAA group during the Al-Nafrah movement, while t our study used more than 800 buses to successfully transport the TEAA group.
- Study [39] simulated the Al-Nafrah movement and indicated a total duration of 7.27 h, whereas our study showed that this task is possible in 6 h.
- Study [40] simulated the movements of 66,000 pilgrims using pedestrian routes. Our study simulated different percentages of all pilgrim groups using pedestrian routes, all within the validated durations. However, given the weather conditions and the physical strain imposed by Hajj pilgrimage, we considered walking as individual pilgrim choice and a fallback solution, showing that public transport (if scheduling is followed) can accommodate more pilgrims.
- Study [14] simulated 376,000 pilgrims travelling through the train movement C from Arafat to Muzdalifah. Our study simulated a larger number of pilgrims (618,000) in a similar scenario, Scenario 3 (TM3).
- Study [3] simulated 153,000 pilgrims, study [14] simulated 120,000+ pilgrims, and [36] simulated 58 groups of TEAA. Our study simulated the movements of all Hajj 2019 pilgrims (3 million), which is equivalent to 12,000 groups, including 573 TEAA groups.

## Conclusion

Hajj involves a large number of pilgrims to be transported between the ritual sites within strict site-time boundaries. The transport process in inherently complex because of the sheer size of the task, short time windows to arrive at specific sites and several sites with multiple times in and out requirements. The movement of pilgrims also involves massive planning and coordination between the heterogeneous modes of transport, with complex scheduling, execution, and replanning if needed. Despite meticulous organisation and experience accumulated over years, Hajj management authorities face problems in transport management, such as late replanning and cascading effects of any delays.

As [8] recently pointed out, Hajj transport management and TEs need to work together to distribute the pilgrims by transport modes, with constant (on the ground) observation/monitoring of the transport operations (e.g., tracking vehicles and pilgrims while boarding, alighting, departing, arriving at sites, walking).

Based on Hajj 2019 data, we have developed and validated several simulation modules for transport and tested several transport scenarios using the DES platform “ExtendSim”. The work presented here aimed at identifying transport problems during the first two days of Hajj, which are most intense in pilgrim movements, and offering potential practical solutions without increasing the fleets or the crowding of the transport. The modelling was modular to allow for coupling of the components, as well as focusing on one transport aspect and movement at a time. The first day of transport from Makkah to Mina is included in a single transport module. For the second day, we developed two modules for transport from two origins (Makkah and Mina) to Arafat then from Arafat to Muzdalifah, including all available transport modes.

The developed transport scenarios addressed some of the issues identified in the literature (congestion, delays, crowding), which if addressed, could pre-empt possible outcomes of the current transport operations and improve Hajj transport management. We relied on using the planned resources and changing the number of pilgrims by their transport mode and time window. In these scenarios, two primary factors were tested; the demand (pilgrim group numbers) and supply (their transport timetables and allocated vehicles), to assess the implications for the services in terms of the adequacy of the fleet to complete the task. Changing the timetables resulted in better results than the scenarios wherein the allocation of pilgrims was changed to the four transport modes. Therefore, we expect that organising group timetables could lead to better transport management outcomes for Hajj. By applying these measures, the time required for transport can be shortened (by more than 12 h, depending on the scenario) compared to the real-time window currently allowed. Additionally, a dynamic reallocation of the conventional and shuttle bus fleets proportional to the number of pilgrims would reduce the average duration of transport while using the fleets more efficiently. These time reductions not only represent savings in the Hajj resources, but also lead to better experience of the pilgrims during their Hajj. This is a critical aspect, given the physical effort required during the five days of Hajj. Considering the heterogeneity of pilgrims (gender, age, cultural diversity) and the presence of pilgrims with mobility difficulties, any additional resting time created by reducing the transport duration would enhance the quality of the experience for all participants and create buffers to ensure start-on-time for rituals that follow. However, the scenario results assume that the scheduled activities are followed as planned, avoiding delays and incidents.

Our validated modules and scenarios simulated 2.6 million pilgrims, which represents the typical attendance in Hajj before the COVID-19 led pandemic and a much higher number than some of the published studies. Managerial strategies were proposed to overcome some issues highlighted in the scenarios, wherein not all allocated pilgrim groups could be transported within the set time window. Distributing the current bus fleet in a more balanced way and increasing the number of pilgrims using the train would ensure the transport capacity, even when weather may prevent transport on foot. For future work, additional transport modules for reverse movement from Muzdalifah to Mina and Mina to Makkah can be coupled to an all-encompassing transport model. Similarly, joining the ritual models with the corresponding before-and-after transport modules makes possible a complete integration of the Hajj activities in a single overall model.

## Supporting information

S1 Table Planned bus durations vs actual bus durations from Hajj 2019. (DOCX)

S2 Table ExtendSim blocks used in the modules [48, 49]. (DOCX)

S3 Table Pilgrim speeds at Hajj event [50]. (DOCX)

S4 Table Conventional bus operations from Makkah to Mina on the 1^st^ day. (DOCX)

S1 Fig Transport module (TM) 1. (DOCX)

S2 Fig TM 1 blocks. (DOCX)

S3 Fig TM1 example equations block. (DOCX)

S4 Fig Transport module (TM) 2. (DOCX)
