# Supplementary material for: Transport of pilgrims during Hajj: Evidence from a discrete event simulation study
Source: PLoS One. 2023 Jun 8;18(6):e0286460. doi: 10.1371/journal.pone.0286460 (PMC10249829; doi:10.1371/journal.pone.0286460)
Supplement: S1 Table — (DOCX) [file pone.0286460.s001.docx]

**S1 Table** - Planned bus durations vs Actual bus durations from Hajj 2019

| **Group** | **Movement** | **From**  **(Date and time)** | **To**  **(Date and time)** | **Transport durations (Hours)** | **Difference (Actual-Planned) (Hours)** |
| --- | --- | --- | --- | --- | --- |
| SEA | Makkah-Mina (Planned) | 7/08/2019 18:00 | 8/08/2019 2:00 | 8 | +1 |
|  | Makkah-Mina (Actual) | 7/08/2019 18:00 | 8/08/2019 3:00 | 9 |  |
|  | Makkah-Arafat (Planned) | 8/08/2019 12:00 | 9/08/2019 3:00 | 15 | +14 |
|  | Makkah-Arafat (Actual) | 8/08/2019 5:00 | 9/08/2019 10:00 | 29 |  |
|  | Mina-Arafat (Planned) | 9/08/2019 6:00 | 9/08/2019 10:00 | 4 | +1 |
|  | Mina-Arafat (Actual) | 9/08/2019 5:00 | 9/08/2019 10:00 | 5 |  |
|  | | | | | |
| Iran | Makkah-Mina (Planned) | 8/08/2019 18:00 | 9/08/2019 0:00 | 6 | -2 |
|  | Makkah-Mina (Actual) | 8/08/2019 18:00 | 8/08/2019 22:00 | 4 |  |
|  | Makkah-Arafat (Planned) | 8/08/2019 17:00 | 9/08/2019 1:00 | 8 | +1 |
|  | Makkah-Arafat (Actual) | 8/08/2019 17:00 | 9/08/2019 2:00 | 9 |  |
|  | Mina-Arafat (Planned) | 9/08/2019 6:00 | 9/08/2019 11:00 | 5 | -1 |
|  | Mina-Arafat (Actual) | 9/08/2019 6:00 | 9/08/2019 10:00 | 4 |  |
|  | | | | | |
| Africa | Makkah-Mina (Planned) | 8/08/2019 1:00 | 8/08/2019 11:00 | 10 | +5 |
|  | Makkah-Mina (Actual) | 7/08/2019 19:00 | 8/08/2019 10:00 | 15 |  |
|  | Mina-Arafat (Planned) | 9/08/2019 7:00 | 9/08/2019 10:00 | 3 | +7 |
|  | Mina-Arafat (Actual) | 8/08/2019 22:00 | 9/08/2019 8:00 | 10 |  |
|  | | | | | |
| SA | Makkah-Mina (Actual) | 8/08/2019 6:00 | 8/08/2019 11:00 | 5 | N/A |
|  | Makkah-Arafat (Actual) | 9/08/2019 7:00 | 9/08/2019 9:00 | 2 |  |
|  | Mina-Arafat (Actual) | 9/08/2019 1:00 | 9/08/2019 11:00 | 10 |  |
|  | | | | | |
| Arabs | Makkah-Mina (Actual) | 7/08/2019 16:00 | 8/08/2019 11:00 | 30 | N/A |
|  | Makkah-Arafat (Actual) | 8/08/2019 18:00 | 8/08/2019 9:00 | 15 |  |
|  | Mina-Arafat (Actual) | 8/08/2019 21:00 | 9/08/2019 8:00 | 11 |  |
|  | | | | | |
| Locals | Makkah-Mina (Actual) | 8/08/2019 12:00 | 9/08/2019 00:00 | 12 | N/A |
|  | Mina-Arafat (Actual) | 8/08/2019 9:00 | 8/08/2019 20:00 | 11 |  |

Note: Data on planned timetables for TEs Locals, SA, Arabs were not provided, therefore table provides only the actual times.
