# Supplementary material for: Transport of pilgrims during Hajj: Evidence from a discrete event simulation study
Source: PLoS One. 2023 Jun 8;18(6):e0286460. doi: 10.1371/journal.pone.0286460 (PMC10249829; doi:10.1371/journal.pone.0286460)
Supplement: S2 Table — (DOCX) [file pone.0286460.s002.docx]

**S2 Table** - Extendsim Blocks Used in the Modules ‎[49,50‎]

| **Block** | **Function** |
| --- | --- |
| Executive Block | Controls the simulation timing and passing of the systems through the module. This block is placed on the left of all other blocks in the modules. The main functions of this block are scheduling events (system processes), control of the simulation, allocating the items, and managing the main attributes. |
| Create Block | Used to generate items (simulated pilgrims or transport modes) randomly or according to fixed schedules. |
| Queue Block | Used as a holding area, wherein items (pilgrims or transport modes) queue up and wait to be processed. The queue releases the items based on their queuing settings. |
| Set Block | Used to sets the properties of items that pass through the block. The settings were pilgrims in groups of 250 based on their establishment. |
| Get Block | Used to produce the properties from items that are passing through. |
| Select In/Out Blocks | Used for merging/separating the flows of the items from a process (input) to another process (output). |
| Transport Block | Used to transport items from one point to another depending on distance and speed. |
| Batch/Unbatch Blocks | Used to combine specified number of inputs into a single output and unbatch a single input to a chosen number of outputs. |
| Resource Pool Block | Used to hold a specified number of capacity units (e.g., buses and trains) for items (pilgrims) passing through the block. |
| Shift Block | Generates schedule over time to be used in the module. |
| Exit Block | Used to pass items out of the simulation. |
| Plotter Block | Used to display information regarding the module performance (throughput, queue lengths, times). |
