# Supplementary material for: Transport of pilgrims during Hajj: Evidence from a discrete event simulation study
Source: PLoS One. 2023 Jun 8;18(6):e0286460. doi: 10.1371/journal.pone.0286460 (PMC10249829; doi:10.1371/journal.pone.0286460)
Supplement: S3 Table — (DOCX) [file pone.0286460.s003.docx]

**S3 Table** - Pilgrim speeds at Hajj event ‎[51]

| **Speed/Velocity (m/s)** | **Category** | **Fitness** | **Age (years)** |
| --- | --- | --- | --- |
| 1.46 | Africa and Asia | Fit | 10-50 |
| 1.2095 |  |  | 50+ |
| 1.3115 |  | Tired | 10-50 |
| 1.0885 |  |  | 50+ |
| 1.1658 |  | Very tired | 10-50 |
| 0.9676 |  |  | 50+ |
| 1.3247 | North America, South America, and Europe | Fit | 10-50 |
| 1.0995 |  |  | 50+ |
| 1.1923 |  | Tired | 10-50 |
| 0.9896 |  |  | 50+ |
| 1.0598 |  | Very tired | 10-50 |
| 0.8796 |  |  | 50+ |
