# Supplementary material for: Transport of pilgrims during Hajj: Evidence from a discrete event simulation study
Source: PLoS One. 2023 Jun 8;18(6):e0286460. doi: 10.1371/journal.pone.0286460 (PMC10249829; doi:10.1371/journal.pone.0286460)
Supplement: S4 Table — (DOCX) [file pone.0286460.s004.docx]

**S4 Table** - Conventional Bus Operations from Makkah to Mina on the 8th

| **Bus station** | **Near to gate of the Grand Masque** | **Distance from the bus station to gate (by walking)** | **Main road/street from the bus stations of The Grand Mosque to Mina** | **Distances from bus stations of the Grand Mosque to pilgrims’ groups' camps at Mina** | **Pilgrim groups using bus station** |
| --- | --- | --- | --- | --- | --- |
| **Ajyad road** | King Abdulaziz Gate | 1 km/ 13 min | King Fahad road/tunnel Rd. 68 | 10 km/18 min | South Asia |
|  |  |  | King Khaled road/tunnel | 11.6 km/28 min |  |
|  |  |  | 3^rd^ ring road and Muzdalifah road | 13 km/38 min | Locals |
| **Ibraheem Al-Khalil Road** | King Fahad Gate | 800 m/12 min | Rd. 68 | 11.2 km/ 35 min | Arab |
|  |  |  | Rd. 38 | 15.3 km/ 39 min |  |
| **Ibraheem Al-Kalil Road 2 (beside Al-Shabekah Graveyard)** | Umrah Gate | 300 m/ 6 min | Rd. 68 | 12.6 km/ 32 min | South-East Asia |
|  |  |  | Route 40-route 80- 4^th^ ring road | 17.6 km/29 min |  |
| **SAPTCO – Northwest The Grand Mosque** | King Abdullah Gate | 920 m/ 14 min | Rd. 68 | 9.8 km/ 22 min | Africa |
|  |  |  | Route 40-route 80- 4^th^ ring road | 10.7 km/ 35 min |  |
| **SAPTCO – Northeast the Grand Mosque** | Al-Fatah Gate | 1.1 km/ 14 min | Rd. 68 | 9.6 km/ 20 min | TEAA |
|  |  |  | Route 40-route 80- 4^th^ ring road | 14.1 km/ 25 min |  |
|  |  |  | Al-Majid Al-Haram Road and Rd. 68 | 13.9 km/ 38 min |  |
|  |  |  | Route 15 | 18.2 km/ 36 min |  |
| **Mina Transit station** | Al-Marwah area Gate | 256 m/ 4 min | Rd. 68 | 9.7 km/ 18 min | Iranian |
|  |  |  | Al-Majid Al-Haram Road and Rd. 68 | 10.5 km/ 30 min |  |

Note: Buses routes are available on Google Map
