# Supplementary material for: Transport of pilgrims during Hajj: Evidence from a discrete event simulation study
Source: PLoS One. 2023 Jun 8;18(6):e0286460. doi: 10.1371/journal.pone.0286460 (PMC10249829; doi:10.1371/journal.pone.0286460)
Supplement: S1 Fig — (DOCX) [file pone.0286460.s005.docx]

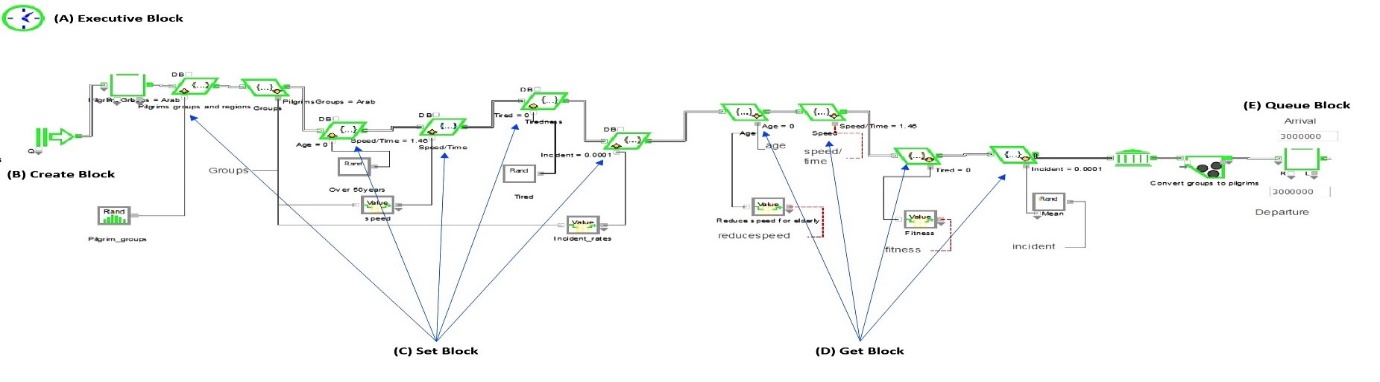


**S1 Fig** - Transport Module (TM) 1 represents the movement of pilgrims from Makkah to Mina on the 8^th^ of Zil Hijjah

(A) Executive, (B) Create, (C) Set, (D) Get, and (E) Queue blocks
