# Supplementary figures and images for: Transport of pilgrims during Hajj: Evidence from a discrete event simulation study
Source: PLoS One. 2023 Jun 8;18(6):e0286460. doi: 10.1371/journal.pone.0286460 (PMC10249829; doi:10.1371/journal.pone.0286460)

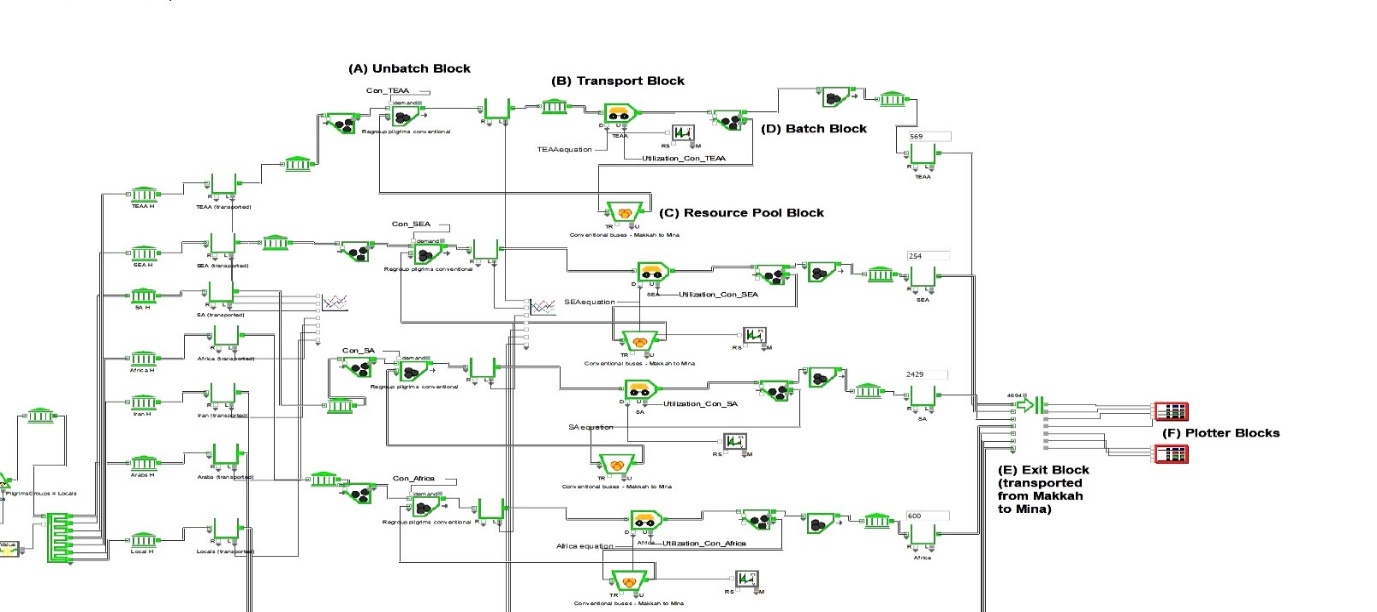


**S2 Fig** - TM1 (A) Unbatch, (B) Transport, (C) Resource, (D) Batch (E) Exit, and (F) Plotter blocks

Supplement: S2 Fig — (DOCX) [file pone.0286460.s006.docx]

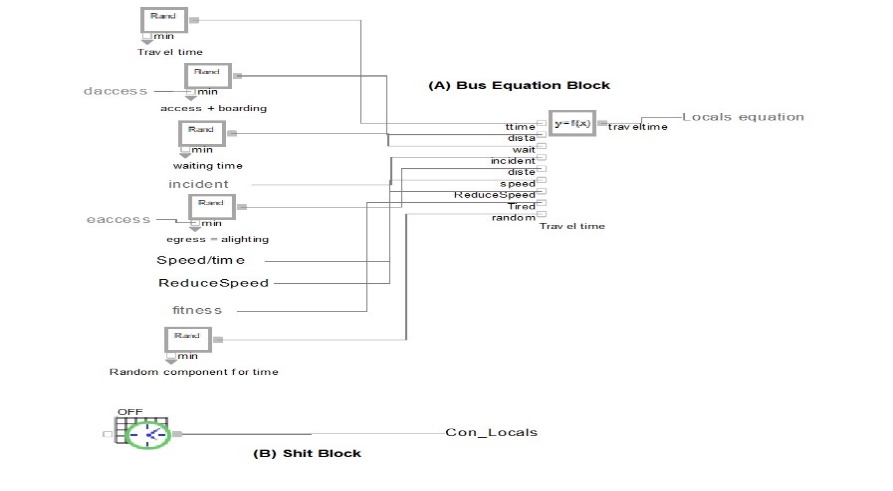


**S3 Fig** - TM1 Example Equations Block

Supplement: S3 Fig — (DOCX) [file pone.0286460.s007.docx]
