# Supplementary material for: Transport of pilgrims during Hajj: Evidence from a discrete event simulation study
Source: PLoS One. 2023 Jun 8;18(6):e0286460. doi: 10.1371/journal.pone.0286460 (PMC10249829; doi:10.1371/journal.pone.0286460)
Supplement: S4 Fig — (DOCX) [file pone.0286460.s008.docx]

| 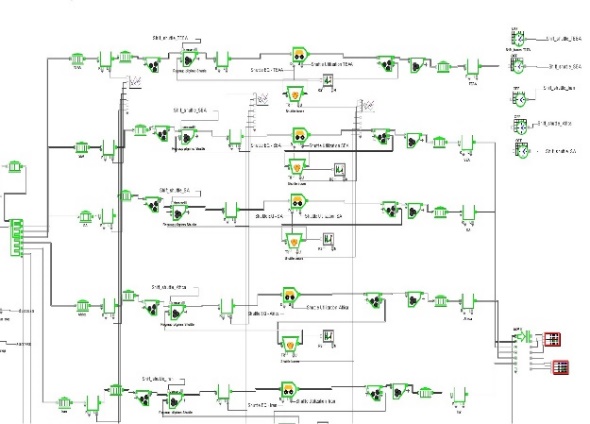  (A) Shuttle buses mode - Mina to Arafat | 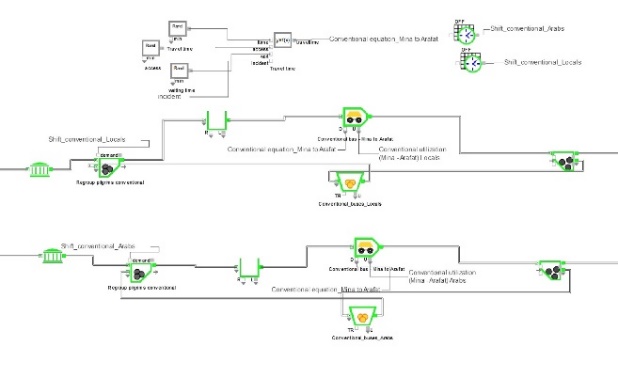  (B) Conventional buses mode - Mina to Arafat |
| --- | --- |
| 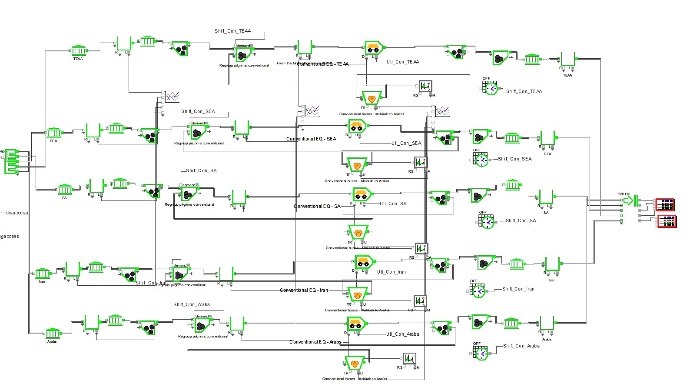  (C) Conventional buses mode - Makkah to Arafat | 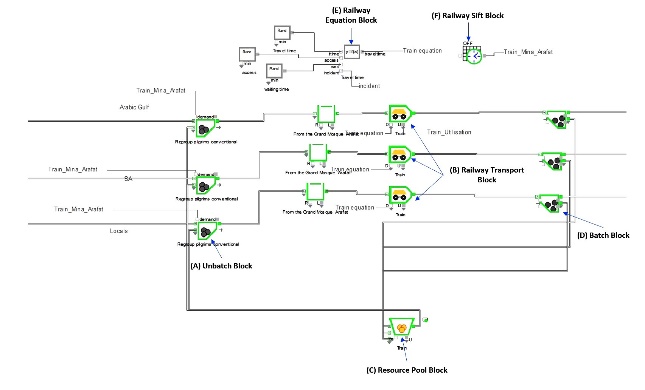  (D) Railway mode |
| 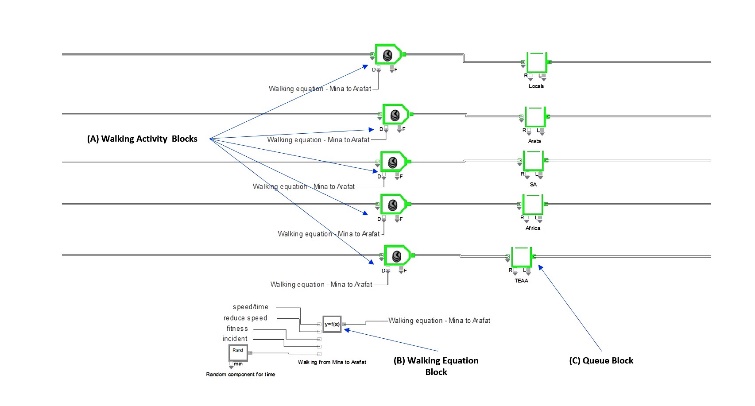  (E) Walking mode | |

**S4 Fig** - Transport Module (TM) 2 represents the movement of pilgrims from Makkah to Arafat (Conventional buses) (C) and Mina to Arafat by, Shuttle buses (A), Conventional buses (B), trains/railways (D) and Pedestrian routes (E).

Note: Models are made available at: <https://cloudstor.aarnet.edu.au/plus/s/aY2LeV4wrDYYc1V>
